# Compositional patterns in the genomes of unicellular eukaryotes

**DOI:** 10.1186/1471-2164-14-755

**Published:** 2013-11-05

**Authors:** Maria Costantini, Fernando Alvarez-Valin, Susan Costantini, Rosalia Cammarano, Giorgio Bernardi

**Affiliations:** 1 Laboratory of Animal Physiology and Evolution, Stazione Zoologica Anton Dohrn, Villa Comunale, Naples 80121, Italy; 2 Sección Biomatemática, Facultad de Sciencias, Universidad de la Repùblica, Montevideo, Uruguay; 3 INT “G. Pascale” - CROM (Oncology Research Centre of Mercogliano), Mercogliano, Italy; 4 Biology Department, Rome 3 University, Viale Marconi 446, Rome, Italy

**Keywords:** Compositional organization, Genome, Unicellular eukaryotes

## Abstract

**Background:**

The genomes of multicellular eukaryotes are compartmentalized in mosaics of isochores, large and fairly homogeneous stretches of DNA that belong to a small number of families characterized by different average GC levels, by different gene concentration (that increase with GC), different chromatin structures, different replication timing in the cell cycle, and other different properties. A question raised by these basic results concerns how far back in evolution the compartmentalized organization of the eukaryotic genomes arose.

**Results:**

In the present work we approached this problem by studying the compositional organization of the genomes from the unicellular eukaryotes for which full sequences are available, the sample used being representative. The average GC levels of the genomes from unicellular eukaryotes cover an extremely wide range (19%-60% GC) and the compositional patterns of individual genomes are extremely different but all genomes tested show a compositional compartmentalization.

**Conclusions:**

The average GC range of the genomes of unicellular eukaryotes is very broad (as broad as that of prokaryotes) and individual compositional patterns cover a very broad range from very narrow to very complex. Both features are not surprising for organisms that are very far from each other both in terms of phylogenetic distances and of environmental life conditions. Most importantly, all genomes tested, a representative sample of all supergroups of unicellular eukaryotes, are compositionally compartmentalized, a major difference with prokaryotes.

## Background

Investigations at the sequence level showed that (i) the genomes of multicellular eukaryotes are compartmentalized in mosaics of isochores that belong to a small number of families that are characterized by different GC levels and dinucleotide frequencies [1-6]. These findings confirmed and extended previous investigations (originally using density gradient ultracentrifugation [7-9]) carried out by our laboratory over many years (see [6] for a review).

The results available so far support the idea of isochores being a “fundamental level of genome organization” [10] not only in vertebrates but also in the other multicellular eukaryotes analyzed. Indeed, as established by our previous work, not only gene distribution, but also chromatin structure, short sequence frequencies, DNA methylation, gene expression, replication timing and recombination are the main structural and functional properties associated with isochore families of all multicellular eukaryotes explored so far. We also proposed that the large conservation of GC levels and dinucleotide frequencies of isochore families reflect the conservation of chromatin structures, whereas the conservation of isochore size might be due to the role played by isochores in chromosome structure and replication [2,11]. These results stress the interest of understanding the structure and the evolution of compositional patterns in unicellular eukaryotes.

Some early results indicated that the nuclear genome of *Euglena gracilis* and the macro-nuclear genome of *Tetrahymena pyriformis* were remarkably homogeneous in base composition, while the nuclear genome of *Saccharomyces cerevisiae* showed a slight heterogeneity [8]. Later work based on sequenced yeast chromosomes showed that some of them consist of alternating large domains of GC-rich and GC-poor DNA [12-14], generally correlating with a variation in gene density. More recent work showed that in yeast GC-rich and GC-poor isochores are different in chromatin conformation, histone modification and transcription; more precisely, GC-rich isochores have a more extended chromatin conformation, different levels of histone acetylation and more highly expressed GC-rich genes [15].

In the case of *Plasmodium falciparum*, the unicellular parasite responsible for the most virulent and widespread form of human malaria, a striking feature is that it hosts the GC-poorest (19.4% GC) nuclear genome known so far [16,17]. In *Plasmodium cynomolgi*, a compositional compartmentalization was demonstrated in the nuclear DNA, which consists of DNA segments likely to average 100 kb [18].

Both DNAs from *Trypanosoma brucei* and *Trypanosoma equiperdum* (two closely related trypanosomes [19,20]) showed a bimodal distribution characterized by two major peaks banding at 1.702-1.703 and 1.707-1.708 g/cm^3^ in CsCl density gradients and representing 1/3 and 2/3 of total DNA, respectively; a number of minor components were also detected, corresponding to satellite DNAs and possibly to ribosomal DNA [21].

In conclusion, the results on yeast, Plasmodia and Trypanosomes indicated that a compositional compartmentalization was not only present in the genomes of metazoan and plants, but also in those of unicellular eukaryotes. These findings encouraged us to extend our investigations to other unicellular eukaryotes.

Other important aspects, indicative of a wide genomic diversity are worth mentioning: 1) The range of genome sizes of unicellular eukaryotes (8.7 Mb to 357 Mb, a 41-fold range; [22]) is even broader than that of metazoans (from 94.4 Mb to 3000 Mb, a 32-fold range, neglecting cases of polyploidy; [4,5]). 2) The range of average GC levels of the genomes of unicellular eukaryotes is as broad as that of prokaryotes [23,24]. 3) The chromatin structure of unicellular eukaryotes may be organized in a different way compared to that of multicellular eukaryotes. For example, *Saccharomyces cerevisiae* lacks histone H1; similarly, Trypanosomes, although they have H1 histone, this protein is quite divergent and chromatin does not reach high levels of compaction during mitosis. 4) The environmental conditions under which unicellular eukaryotes live are much more diverse than those of vertebrates and also of invertebrates. 5) Unicellular eukaryotes lack the very complex regulatory system involved in the developmental process of multicellular eukaryotes.

All these considerations prompted us to tackle the analysis of compositional organization in unicellular eukaryotes. Here we approached these problems by studying the genomes of representative species from all the so-called “supergroups” of unicellular eukaryotes.

## Results

In this work we studied the compositional organization in representative species from all the so called eukaryotic “supergroups” (see also Additional file 1: Table S1 and refs. [25,26]). In Additional file 2: Figure S1 we report the phylogenetic distribution of the unicellular species analyzed in the present work [24].

Green and red algae (*Ostreococcus tauri, Cyanidioschyzon merolae* respectively) represent Plantae. The supergroup Amebozoa is represented by the slime mold, *Dictyostelium discoideum*. In the supergroup Chromoalvelata we analysed species from the four main groups: two diatoms (*Thalassiosira pseudonana* and *Phaeodactylum tricornutum*) representing, *Stramopiles.* For the *Apicomplexans* (that include parasitic species in mammals) we analysed the human pathogen *Toxoplasma gondii* and the malarial parasites *Plasmodium berghei*, *Plasmodium chabaudi*, *Plasmodium knowlesi*, *Plasmodium falciparum* and *Plasmodium vivax*. The Cryptophyta group is represented by *Guillardia theta,* while for the last group, Ciliates, the analysis was only partial due to the fragmented genome assembly that is available for this species. The Excavata supergroup is represented by two Kinetoplastids (*Trypanosoma brucei* and *Trypanosoma cruzi*), while in the Fornicata group, the species analysed was *Giardia lamblia,* even if, in this case too, the analysis was only partial due to the incompleteness of the assembled genome. Finally the supergroup Opisthokonta (which also includes animals) is represented here by several unicellular fungi: *Saccharomyces cerevisiae*, *Candida glabrata*, *Ashbya gossypii* and *Cryptococcus neoformans*.

The different groups of organisms studied here exhibit a diversity of genome compositional patterns, that range from very weak to very strong compartmentalization (see Table 1).

**Table 1:** Average GC (A) and relative amounts (B) in percentage of components from unicellular eukaryotes

**A) Average GC**								
**Algae**	*O. tauri*							59.4^(a)^
	*C. merolae*					51.8	55.4	
**Diatoms**	*T. pseudonana*					47.0		
	*P. tricornutum*					48.9		
**Fungi**	*S. cerevisiae*			38.5				
	*C. glabrata*		35.7	38.9	43.5			
	*A. gossypii*					50.7	53.8	
	*C. neoformans*					48.9		
**Flagellate protozoans**	*T. brucei*				43.2	47.5		
	*T. cruzi*					49.6	54.7	
**Parasitic protist**	*T. gondii*					52.7	55.0	
**Amoeba**	*D. discoideum*	28.4^(a)^						
**Malaria parasites**	*P. falciparum*	19.4^(a)^						
	*P. chabaudi*	22.5^(a)^						
	*P. berghei*	22.0^(a)^						
	*P. knowlesi*		35.0	38.5	42.8			
	*P. vivax*		32.6	39.1	43.7	46.2	53.7	
	**Overall average**		34.2	38.8	43.5	48.8	54.4	
			**L1**	**L2**	**H1**	**H2**	**H3**	
	**Vertebrates**^**(b)**^		36.2	39.1	43.3	48.3	54.8	
	**Invertebrates**^**(b)**^		35.5	39.3	43.0	47.1	54.5	
**B) Relative amount**								
**Algae**	*O. tauri*							~100^(c)^
	*C. merolae*					88.2	10.9	
**Diatoms**	*T. pseudonana*					~100^(c)^		
	*P. tricornutum*				3.6	96.4^(c)^		
**Fungi**	*S. cerevisiae*			95.0	0.7			
	*C. glabrata*		16.0	79.0	5.5			
	*A. gossypii*					60.0	40.0	
	*C. neoformans*					~100^(c)^		
**Flagellate protozoans**	*T. brucei*				18.9	76.7		
	*T. cruzi*					78.8	23.7	
**Parasitic protist**	*T. gondii*					83.0	17	
**Amoeba**	*D. discoideum*	~100^(c)^						
**Malaria parasites**	*P. falciparum*	~100^(c)^						
	*P. chabaudi*	~100^(c)^						
	*P. berghei*	~100^(c)^						
	*P. knowlesi*		7.5			45.5	47.0	
	*P. vivax*		11.7	6.4	37.0	47.8	2.7	

^(a)^These genomes were excluded from the calculation of the overall average because of their extreme values.

^(b)^For the sake of comparison the overall average is reported for isochore families from vertebrate and invertebrate genomes analyzed in Costantini et al. [4] and Cammarano et al. [5].

^(c)^These values included minor components.

**Figure 1:**
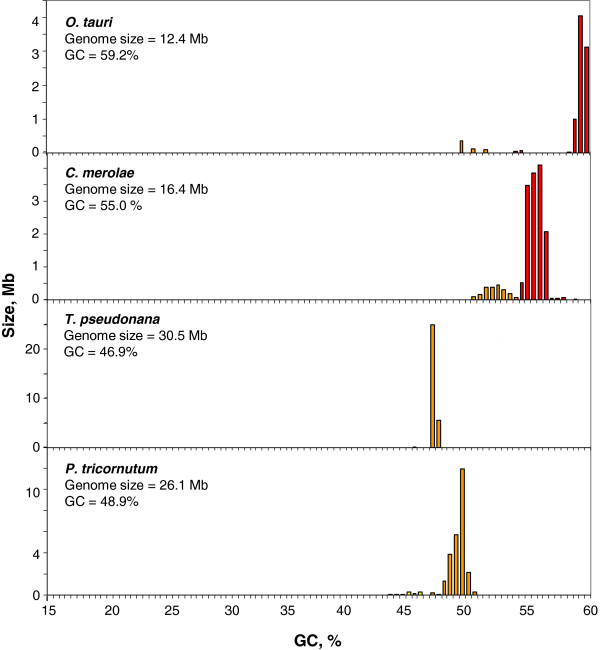
**Distribution by weight of DNA segments according to GC levels in the green alga *O. tauri*, in the red alga *C. merolae *** **and in diatoms *T. pseudonana *** **and *P. tricornutum.***

The results obtained in this work indicate that unicellar eukaryotes encompass a wide range of situation in terms of genomic composition and heterogeneity. In the first group, namely Algae, the green alga *Ostreococcus tauri* and the red alga *Cyanidioschyzon merolae* showed very GC-rich genomes (see Figure 1). In the first case, DNA was centered at 59-60% GC, in the second at 55-56% GC, with a smaller component at 52-53% GC. Both diatoms analyzed, *Thalassiosira pseudonana* and *Phaeodactylum tricornutum*, showed GC-rich genomes consisting of components that were centered at 47% and 49% GC, respectively (Figure 1).

**Figure 2:**
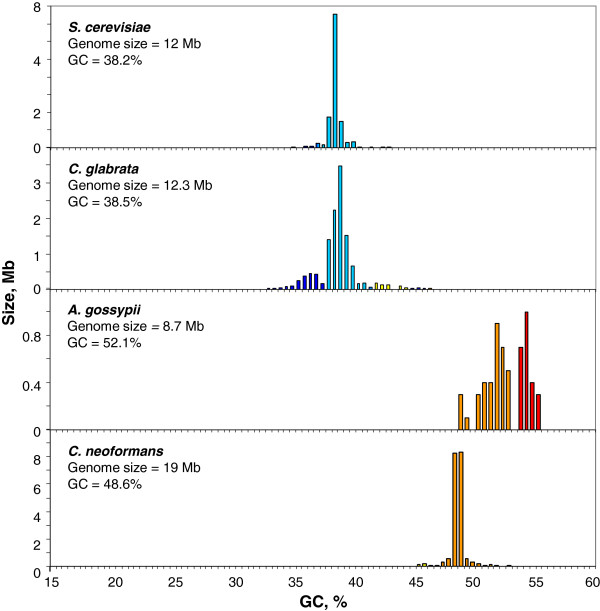
**Distribution by weight of DNA segments according to GC levels in fungi, *S. cerevisiae, C. glabrata, A. gossypii *** **and *C. neoformans.***

The genomes of fungi exhibited very different GC ranges (see Figure 2). Indeed, *Saccharomyces cerevisiae* and *Candida glabrata* showed GC-poor genomes, essentially consisting of DNA components centered at 38-39%, that were accompanied in the case of *C. glabrata* by a minor component ranging from 34% to 38% GC and also by a very minor GC-richer component in the 42-46% GC range. In contrast, the other two fungi analyzed showed GC-richer genomes: *Ashbya gossypii* comprised two GC-rich components, the first one centered at about 52-53% GC, the second one centered at 55% GC, whereas *Cryptococcus neoformans* exhibited one component centered at 48-49% GC.

**Figure 3:**
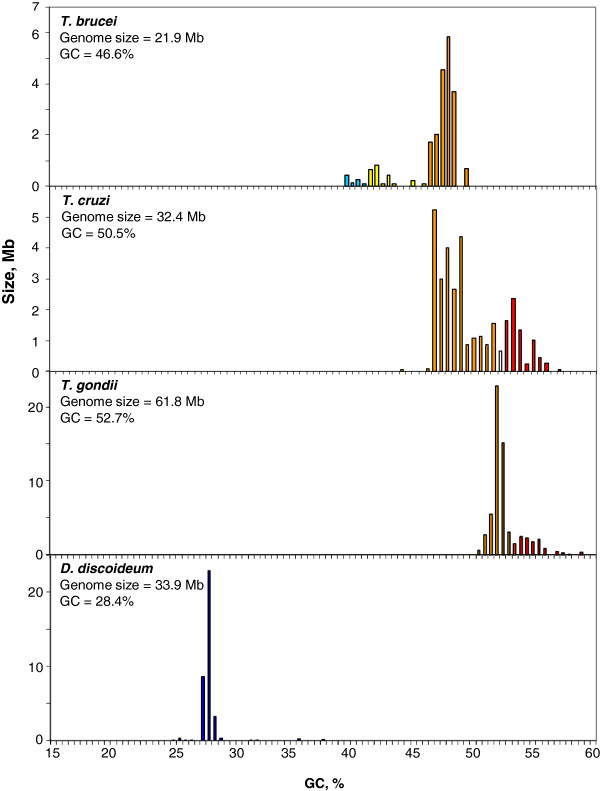
**Distribution by weight of DNA segments in protists *T. cruzi, T. brucei *** **and *T. gondii *** **and *D. discoideum.***

Protists are an exceptionally diverse group from a phylogenetic viewpoint. Indeed, the genome-wide distances and times of divergence between two protozoan groups are many times larger than those of the most divergent metazoans. In this work we have studied species that are representative of all major groups among which two well known groups of human parasites, Trypanosomatids and Plasmodia. As long as the first of these two groups is concerned, it is interesting to note that *Trypanosoma brucei* and *Trypanosoma cruzi*, exhibited GC-rich genomes (Figure 3). In particular the first one was essentially formed by a component centered at 48% GC, and by minor GC-poorer ones; the second one showed two main components, the first one centered at 48% GC, the second, smaller one at 54% GC.

**Figure 4:**
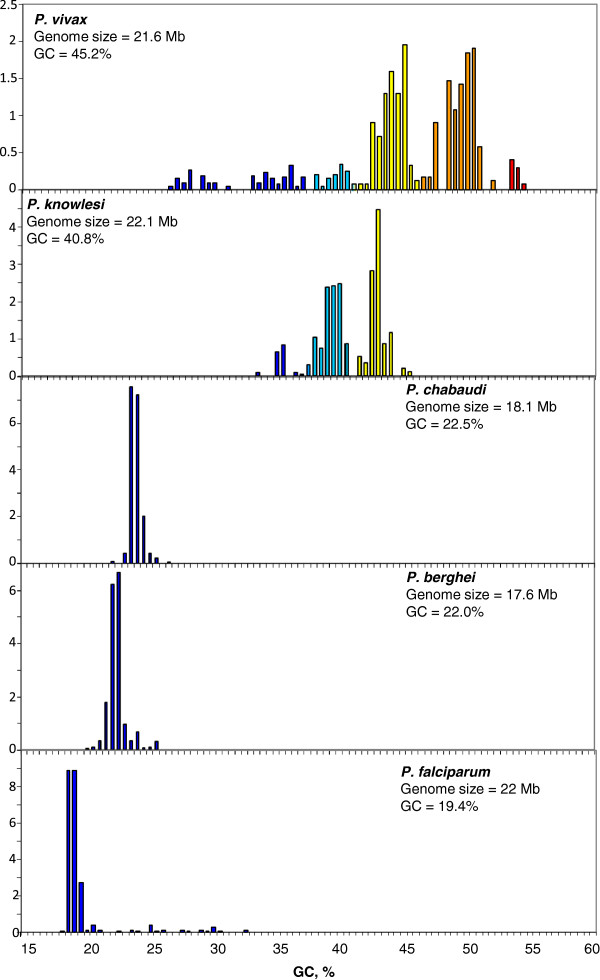
**Distribution by weight of DNA segments in protists *P. vivax*, *P. knowlesi*, *P. chabaudi*, *P. berghei *** **and *P. falciparum.***

As far as the second group is concerned (see Figure 4), the situation is more striking because the malaria parasite *Plasmodium vivax* exhibits a genome covering a broad compositional spectrum (28%-55% GC) with two major components centered at about 44% and 49% GC, whereas in an exceedingly sharp contrast, *Plasmodium chabaudi*, *Plasmodium berghei,* and *Plasmodium falciparum,* which have genome sizes very close to that of *P. vivax*, showed very GC-poor genomes with single major components centered at 24%, 22% and 19.4% GC, respectively. Only the *P. falciparum* genome showed some minor components ranging from 20% to 32% GC. *Plasmodium knowlesi* exhibited a genome pattern which was intermediate between *P. falciparum* and *P. vivax*, exhibiting two major components centered at about 39% and 43% GC as well as a smaller component at 35% GC. All the DNA components from unicellular genomes were grouped in families according to their GC levels, as reported in Table 1.

The parasitic protist *Toxoplasma gondii* consisted of one major component centered at 52% GC and a smaller component at 55% GC, whereas the Amoeba *Dictiostelium discoideum* showed one major component centered at 28% GC (Figure 3).

**Figure 5:**
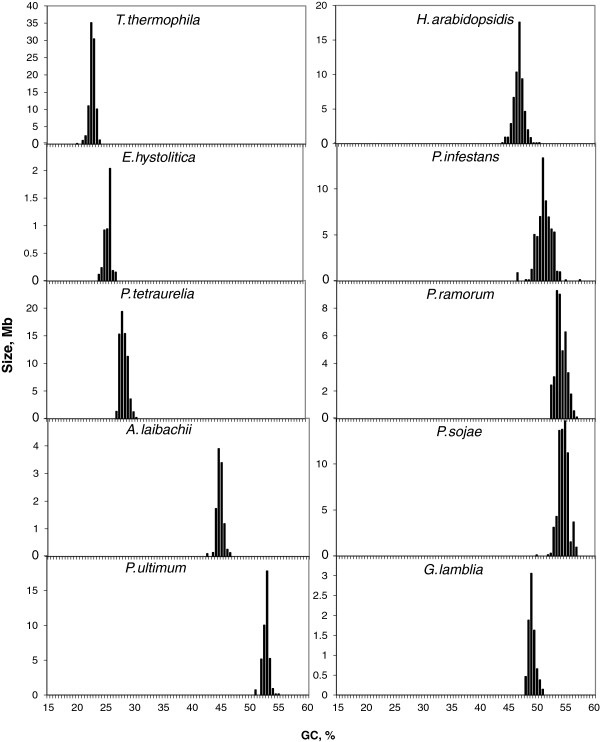
**The amounts of DNA in megabases (Mb) for contigs/scaffolds of unicellular genomes listed in Table**2 **pooled in bins of 0.5% GC.**

Unfortunately, only contigs/scaffolds were available for the genomes of the unicellular eukaryotes listed in Table 2 (see Additional file 2: Figure S1). In these cases, we analysed the contigs/scaffolds larger than 100 kb that represented a large percentage of the available sequences as shown in Additional file 3: Table S2. Several of these genomes covered some missing taxa (at the group classification level, see Additional file 2: Figure S1), such as ciliates, while others belong to taxa for which a complete analysis was done in other species from the same group (like Stramopiles). These genomes covered a wide GC spectrum, ranging from the very GC-poor genome for *Tethahymena thermophila* to the very GC-rich genome of *P. sojae* (as reported in Figure 5).

**Table 2:** GC content, number of contigs/scaffolds and their total lengths in megabases (Mb), length of scaffolds > 100 and > 100 kb and their percentage on the total length were reported

**Unicellulars**	**GC, %**	**Contigs/Scaffolds**	**Size_tot**	**< 100 Kb**	**> 100 Kb**	**< 100 Kb**	**> 100 Kb**
		**(number)**	**(Mb)**	**(Mb)**	**(Mb)**	**(%)**	**(%)**
***Tethahymena_thermophila***	21.1	1158	103.0	10.0	92.9	9.7	90.3
***Entamoeba_hystolitica***	22.7	1529	20.8	16.0	4.8	76.9	23.1
***Paramecium_tetraurelia***	27.5	696	71.2	3.5	67.7	4.9	95.1
***Albugo_laibachii***	44.5	3827	32.7	21.8	10.9	66.8	33.2
***Phytium_ultimum***	46.0	975	42.8	2.5	40.3	5.9	94.1
***Hyaloperenospora_arabidopsidis***	48.4	3044	70.8	14.0	56.8	19.8	80.2
***Phytophtora_infestans***	52.1	4887	89.4	28.0	61.4	31.3	68.7
***Phytophtora_ramorum***	53.6	2576	54.4	13.7	40.7	25.2	74.8
***Phytophtora_sojae***	53.9	1810	78.1	10.7	67.4	13.7	86.3
***Giardia_lamblia***	54.3	306	11.2	3.0	8.2	26.6	73.4

The sequences of these genomes were downloaded from Ensembl website.

**Figure 6:**
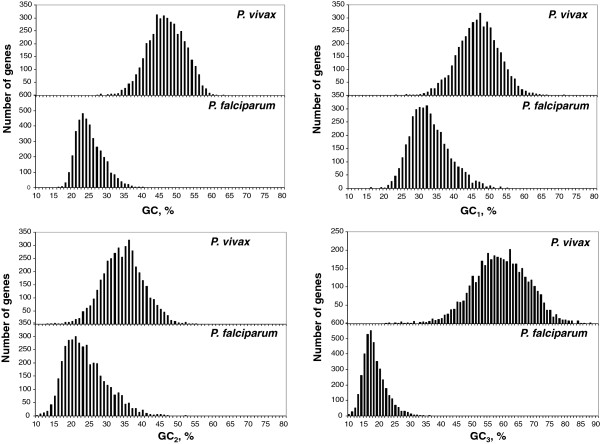
**The histograms show the distributions of the GC, GC**_**1**_**, GC**_**2 **_**and GC**_**3 **_**for the coding sequences of a set of about 4000 orthologous genes for *P. vivax *** **and *P. falciparum*.**

**Figure 7:**
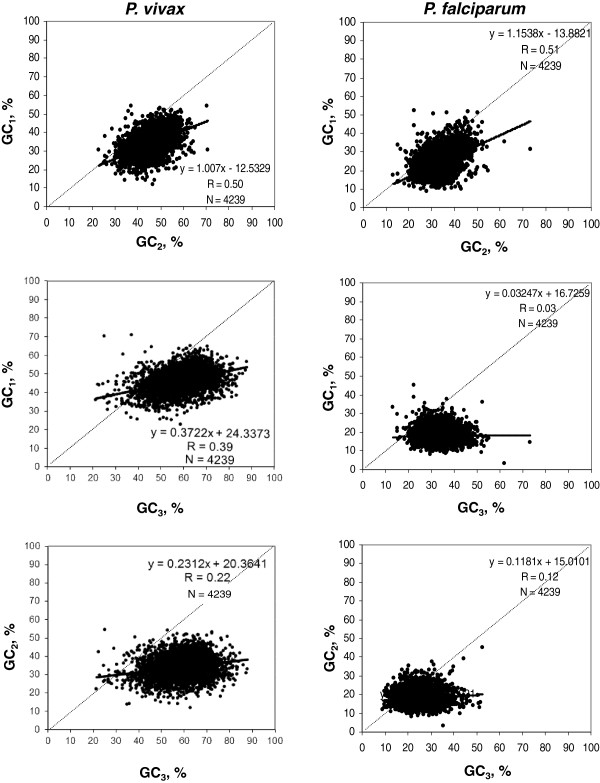
**Scatterplots of GC, GC**_**1**_**, GC**_**2 **_**and GC**_**3 **_**among themselves for orthologous genes for *P. vivax *and *P. falciparum*.** The orthogonal regression equations, the correlation coefficient (R) and the number of genes (N) are reported. The main diagonal is indicated by a broken line.

The extreme contrast between the compositional patterns of *P. vivax* and *P. falciparum* prompted us to analyze (using about 4,000 orthologous genes) the compositional distribution of GC, GC_1,_ GC_2 ,_ GC_3_ as well as the correlations between the GC levels of the three codon positions. The first analysis (Figure 6) showed, as expected, a strong shift towards lower values of the distributions from *P. vivax* to *P. falciparum*, reaching a complete absence of overlap in the case of GC_3._ The second analysis (Figure 7) showed a very significant correlation coefficient 0.50-0.51, for the GC_1_*vs.* GC_2_, as expected from the universal correlation of D’Onofrio and Bernardi [27]. In contrast, the correlations between GC_1_/GC_2_ and GC_3_ were weaker in *P. vivax* (0.39 and 0.22, respectively) and very weak or absent in *P. falciparum* (0.03 and 0.12, respectively), a result likely to be linked to the extremely low values and narrow distribution of GC_3_.

## Discussion

The results just reported clearly show that the genomes of unicellular eukaryotes range from narrow compositional distributions, as in the case of *O. tauri*, *T. pseudonana*, *C. neoformans* and *P. falciparum*, *P. berghei* and *P. chabaudi*, to more heterogeneous patterns, such as those of *S. cerevisiae* and *T. brucei,* while in many other groups such as *P. knowlesi*, *P. vivax*, and *T. cruzi,* the heterogeneity is remarkable. These observations deserve some general comments (in addition to those already made in the preceding section).

Several findings are very striking when compared with both vertebrate and invertebrate genomes. Even if the number of genomes is admittedly modest, a first observation is that free-living unicellular organisms generally show narrower compositional distributions with only minor additional components (*S. cerevisiae* and *A. gossypii*, the latter showing, however, a slightly wider compositional range; 52%-55% GC). This narrow distribution is centered, however, on very different GC levels, that range from 38%-40% GC for the two yeasts to almost 60% GC for the green alga *O. tauri*. Obviously, it would be interesting to correlate these very different compositions to environmental factors. This seems, however, to be possible only for *C. merolae,* in which case the high GC level (55% GC) might be related to the hot acid springs (45°C; pH 2.0) of its habitat. This idea is supported by our previous findings in which high GC levels are correlated with the high body or optimal growth temperatures, in the case of vertebrates and bacteria, respectively (see [6] for a review). Interestingly, protein divergence between *Galdieria sulphuraria*, which lives like *C. merolae* in hot spring, and *Galdieria phlegrea*, which lives in less extreme habitat (*i.e.* moderate pH and temperature) is similar to that between human and medaka [28].

In contrast, parasitic unicellular organisms show some striking features, namely that within the same genus one species may have a wide compositional distribution (this is the case of *T. cruzi* and of *P. vivax*) and other ones have a very narrow distribution (*P. falciparum, P. berghei* and *P. chabaudi*). These results are highly suggestive of compositional adaptation. Needless to say, it would be of great interest to identify the causes for such adaptations, especially since recent results [29] reported a lack of synteny among Apicomplexa due to genome rearrangements.

The compositional compartmentalization of some genomes of unicellular eukaryotes is possibly linked to a different chromatin structure and different regulation of gene expression. The results of Table 1 also show something of great potential interest, namely that, apart from the extreme cases of *P. falciparum* and *O. tauri,* the GC values for the single or multiple DNA components are very close to those previously found for the isochore families of vertebrates and invertebrates. This might be a coincidence, but might also be linked to specific features of chromatin structures. Needless to say that it would be also very interesting to consider whether genes characterized by specific functions are differentially distributed in the two major families exhibited by *T. brucei* and *P. vivax*, respectively.

At this point, it is worthwhile mentioning that an intrachromosomal compositional heterogeneity was also found in prokaryotic genomes [30]. In fact, while most prokariotic species tested are compositionally homogeneous, a minority are rather heterogeneous in composition, an explanation, being, however, associated with recent lateral transfers.

## Conclusions

Previous results on the genomes from a small number of unicellular eukaryotes provided the first indication that a compositional compartmentalization was not only present in the genomes of multicellular eukaryotes, but also in those of some protozoa. The findings presented here revealed that situations of compositional compartmentalization covering a very broad range were generally present in unicellular eukaryotes. Even if the sample of organisms investigated is admittedly modest this point is clearly demonstrated. This distinguishes eukaryotes that always show compartmentalized genomes from prokaryotes, in which case the compositional heterogeneity is exceedingly rare and possibly always associated with recent lateral tranfers.

The results presented here, and previous observations (like those already mentioned for the budding yeast), lead us to suggest that genome compartmentalization is a very general feature of all eukaryotes. Different levels of compartmentalization are probably linked with increasing regulatory complexity and/or other functional requirements to which organisms are bound. This idea is in line with a more general notion in Biology concerning the role of compartmentalization as a fundamental way to organize structure and function at all levels from the organ level down to the cellular and genome level.

Two additional conclusions we consider as preliminary, but, if confirmed by investigations on a larger sample, would be of very great interest. The first one concerns the differences found between free-living and parasitic unicellular eukaryotes. The second one, the fact that GC levels found in unicellular eukaryotes are very close (with two exceptions) to those of isochore families from multicellular eukaryotes. Indeed, the first point suggests compositional adaptation of the genomes of parasitic unicellular organisms, the second a correlation with chromatin structure.

## Methods

### Genome and gene sequences: the resources

The sequences of unicellular genomes as well as those of the genes analyzed in this study were downloaded from different websites (see Additional file 3: Table S2). Partial, putative, synthetic construct, predicted, not experimental, hypothetical protein, r-RNA, t-RNA, ribosomal and mitochondrial genes were eliminated and then the cleanup program [31] was applied for ridding nucleotide sequence databases of redundancies. For the remaining genes a script implemented by us was used in order to identify the coding sequences beginning with a start codon and ending with a stop codon. The coordinates of genes on the chromosomes were retrieved from the website used for downloading the chromosomes.

### Compositional patterns: methodology and nomenclature

The entire chromosomal sequences of the finished genome assembly were partitioned into non-overlapping windows, and their GC levels were calculated using the program draw_chromosome_gc.pl [32,33]. The general methodology used to map DNA segments on unicellular genomes was that described for the isochore map of vertebrates [1] and invertebrates genomes [4,5]. It should be stressed that this methodology has a trend to overestimate compositionally homogeneous regions, because the standard deviation tends to decrease with increasing size of the regions. Because of the small chromosome sizes of several unicellular genomes under analysis, we used a non-overlapping window of 25 kb, a size suitable for all the unicellular genomes. The GC levels of compositionally nearly homogeneous DNA segments were calculated using a script implemented by us. The sequences of contigs/scaffolds for unicellular genomes reported in Table 2 were downloaded from Ensembl Genome Browser (http://protists.ensembl.org/).

In order to demonstrate that the different compositional patterns found were not an artifact due to the small window used (25 kb), we analyzed two unicellular genomes showing a strong compositional heterogeneity using two different non-overlapping windows. Additional file 4: Figure S2A-B display the compositional profiles of *T. brucei* and *P. vivax* at windows of 25 kb and 100 kb. The results clearly demonstrate that the levels of heterogeneity at 25 kb were barely larger than at 100 kb.

Additional file 5: Tables S3-S20 report the coordinates, sizes and GC levels of the segments identified in the genomes. When these segments were pooled in bins of 0.5% GC, families of segments were found according to their average GC levels. Table 1 reports the average GC levels and the relative amounts from these families. For the sake of comparison, Table 1 also shows the average GC levels calculated for the different isochore families of vertebrates [4] and invertebrates [5].

As far as the name of each DNA segment is concerned we used a convention in which the first number in the name represents the chromosome number, the following two letters are the initials of the scientific name of the species under consideration, and the last number identifies the fragment.

## Competing interests

The authors declare that there are no competing interests.

## Authors’ contributions

MC- Conceived the research, identified and mapped the components on the chromosomes from unicellular genomes, performed the compositional analysis on orthologous genes for *Plasmodium* and drafted the manuscript. FAV- Contributed mainly to the analysis of Tripanosomes, helped to the analysis of data and to the drafting of the manuscript. SC- Contributed in bioinformatics analysis of data. RC- Contributed in performing the analysis of data and in calculating gene density. GB- Designed the research and wrote the paper. All contributed to the writing of the manuscript, and all read and approved the final manuscript.

## Supplementary Material

Additional file 1: Table S1 Classification of unicellular species according reference Genomes analysed in the present work are classified according references mentioned in Additional file 2: Figure S1. The “v” symbol indicates whether the analysis conducted was partial (due to the complications with the available genome assembly), or complete. The dash indicates that the group was not analysed due to lack of genome sequence data. Click here for file

Additional file 2: Figure S1 Phylogenetic distribution of the unicellular species analyzed in this work. The taxonomic distribution of species is presented according to information provided in references [23,24], the phylogenetic tree is from Katz et al. see ref. [22] with modifications. Red arrows indicated the complete unicellular genomes assembled in chromosomes; blue arrows indicated unicellular genomes assembled in contigs/scaffolds (see also Additional file 1: Table S1). Because of space constraints not all species analyzed in this work are located on the tree. The full listing of species, discriminated by taxonomic group, is provided in Additional file 1: Table S1. Click here for file

Additional file 3: Table S2 Genome websites, genome size, number of chromosomes, average GC for unicellular genomes. Click here for file

Additional file 4: Figure S2 Distribution by weight of DNA segments according to GC levels (A) in *T. cruzi* and (B) in *P. vivax*, considering two non-overlapping windows at 25 kb and 100 kb*.* Click here for file

Additional file 5: Table S3 Coordinates, sizes and GC levels of *O. tauri* segments. **Table S4.** Coordinates, sizes and GC levels of *C. merolae* segments. **Table S5.** Coordinates, sizes and GC levels of *T. pseudonana* segments. **Table S6.** Coordinates, sizes and GC levels of *P. tricornutum* segments. **Table S7.** Coordinates, sizes and GC levels of *S. cerevisiae* segments. **Table S8.** Coordinates, sizes and GC levels of *C. glabrata* segments. **Table S9**. Coordinates, sizes and GC levels of *A. gossypii* segments. **Table S10**. Coordinates, sizes and GC levels of *C. neoformans* segments. **Table S11**. Coordinates, sizes and GC levels of *T. brucei* segments. **Table S12**. Coordinates, sizes and GC levels of *T. cruzi* segments. **Table S13**. Coordinates, sizes and GC levels of *P. falciparum* segments. **Table S14**. Coordinates, sizes and GC levels of *P. vivax* segments. **Table S15**. Coordinates, sizes and GC levels of *T. gondii* segments. **Table S16**. Coordinates, sizes and GC levels of *P. knowlesi* segments. **Table S17**. Coordinates, sizes and GC levels of *P. berghei* segments. **Table S18**. Coordinates, sizes and GC levels of *P. chabaudi* segments. **Table S19**. Coordinates, sizes and GC levels of *G. theta* segments*. **Table S20**. Coordinates, sizes and GC levels of *D. discoideum* segments. Click here for file
