# Supplementary material for: Compositional patterns in the genomes of unicellular eukaryotes
Source: BMC Genomics. 2013 Nov 5;14:755. doi: 10.1186/1471-2164-14-755 (PMC4007698; doi:10.1186/1471-2164-14-755)
Supplement: Additional file 1: Table S1 — Classification of unicellular species according reference Genomes analysed in the present work are classified according references mentioned in Additional file 2: Figure S1. The “v” symbol indicates whether the analysis conducted was partial (due to the complications with the available genome assembly), or complete. The dash indicates that the group was not analysed due to lack of genome sequence data. [file 1471-2164-14-755-S1.pdf]

Additional Table T1. Classification of unicellular species according reference Genomes analysed in the present work are classified according references mentioned in Additional figure 1. The "v" symbol indicates whether the analysis conducted was partial (due to the complications with the available genome assembly), or complete. The dash indicates that the group was not analysed due to lack of genome sequence data.

Supergroup[+]	Group	Species	Genome	
			Complete	Partial assembly
Amoebozoa				
	**Lobose amoebae**	Amoeba	----	------
	**Slime molds**			
		Dictyostelium discoideum	v	
	**Pebionts**			
		Entamoeba hystolytica		v
Chromalveolata				
	**Ciliates**			
		Paramecium tetraurelia		v
		Thetrahymena thermophila		v
	**Apicomplexa**			
		Toxoplasma gondii	v	
		Plasmodium falciparum	v	
		Plasmodium vivax	v	
		Plasmodium knowlesi	v	
		Plasmodium chabaudi	v	
	**Stramenopiles**			
		Albugo laibachii		v
		Phytophora infestants		v
		Phytophora ramorum		v
		Phytophora sojae		v
		Pythium ultimum		v
		Halyoperonospora arabidopsis		v
		Thalasiossira pseudonana (diatom)	v	
		Phaedactilum tricornutum (diatom)	v	
	**Cryptophyta**			
		Guillardia theta		v

Excavata				
	**Fornicata** (Diplomonads)		---	----
		Giardia lamblia		v
	**Euglenozoa (kineoplatids)**			
		Trypanosoma cruzi	v	
		Trypanosom brucei	v	
	**Parabasalis**			
Plantae				
	**Green algae** (includes land plants)			
		Ostreococcus tauri	v	
	**Red algae**	Cyanidioschyzon merolae	v	
	Glaucophytes			
Opisthokonta				
(also includes Animals, i.e metazoa and Choanoflagelata (sponges)	**Fungi**			
		S. cerviseae	v	
		Candida glabrata	v	
		Ashbya gossypii	v	
