# Supplementary figures and images for: Compositional patterns in the genomes of unicellular eukaryotes
Source: BMC Genomics. 2013 Nov 5;14:755. doi: 10.1186/1471-2164-14-755 (PMC4007698; doi:10.1186/1471-2164-14-755)

# Additional File 1

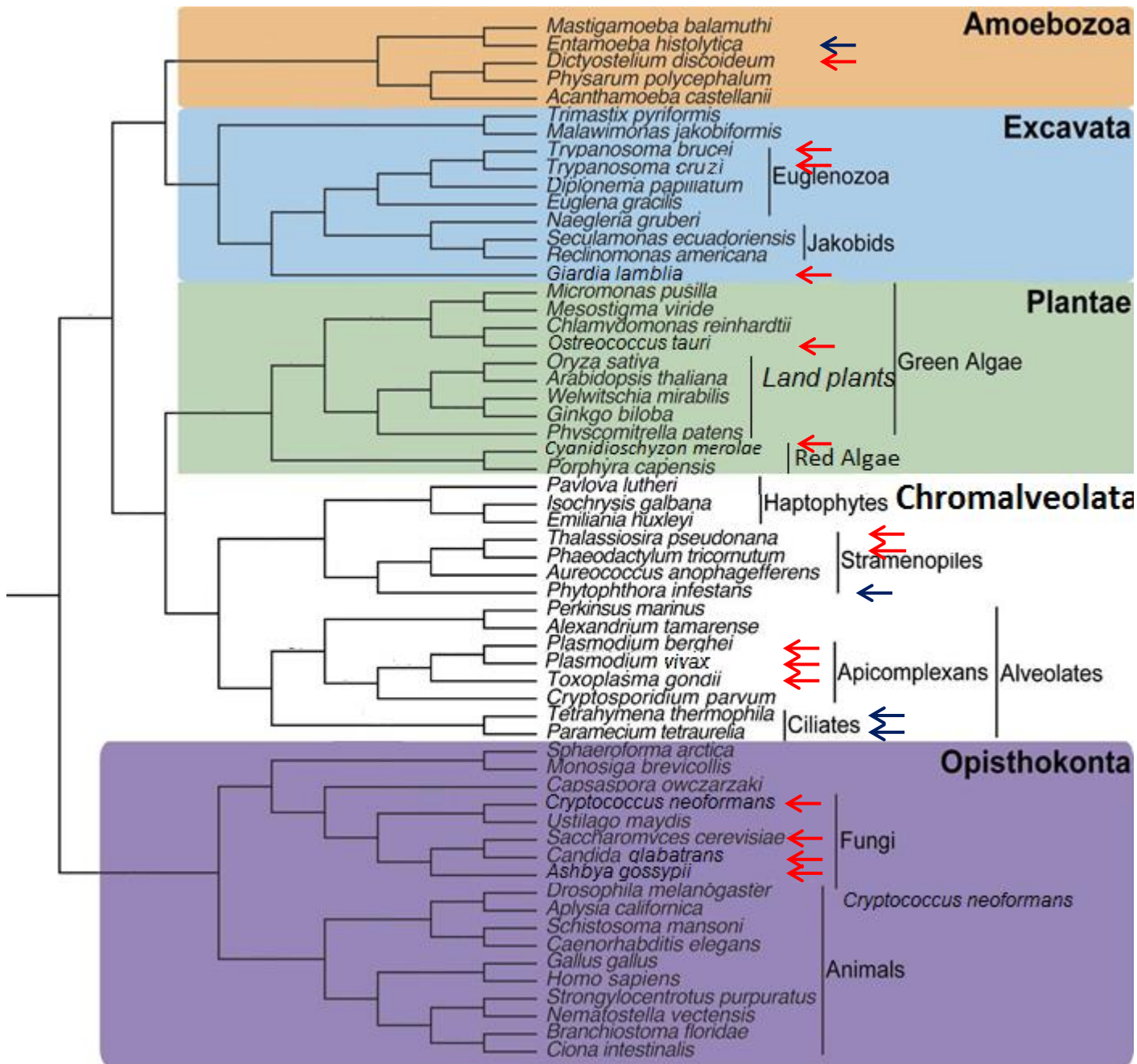

Supplement: Additional file 2: Figure S1 — Phylogenetic distribution of the unicellular species analyzed in this work. The taxonomic distribution of species is presented according to information provided in references [23,24], the phylogenetic tree is from Katz et al. see ref. [22] with modifications. Red arrows indicated the complete unicellular genomes assembled in chromosomes; blue arrows indicated unicellular genomes assembled in contigs/scaffolds (see also Additional file 1: Table S1). Because of space constraints not all species analyzed in this work are located on the tree. The full listing of species, discriminated by taxonomic group, is provided in Additional file 1: Table S1. [file 1471-2164-14-755-S2.pdf]

# Additional file 2

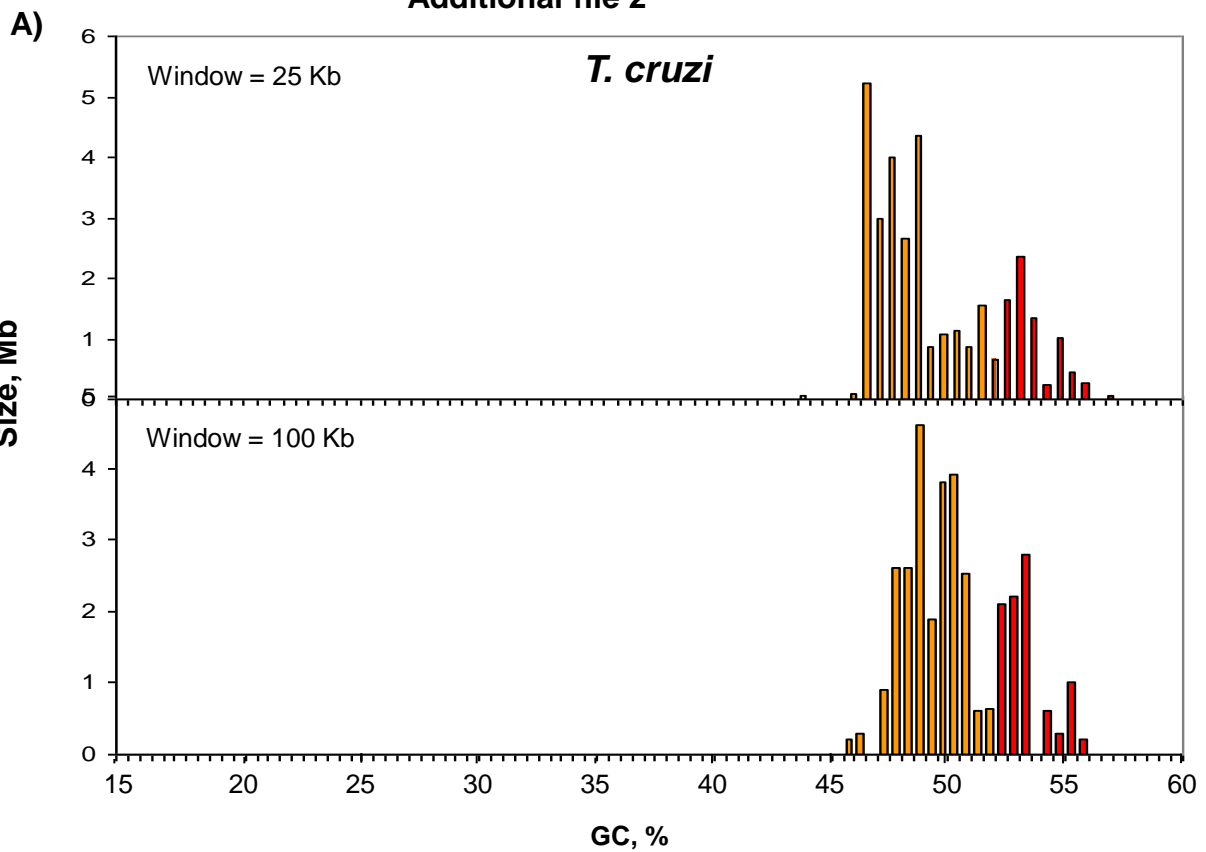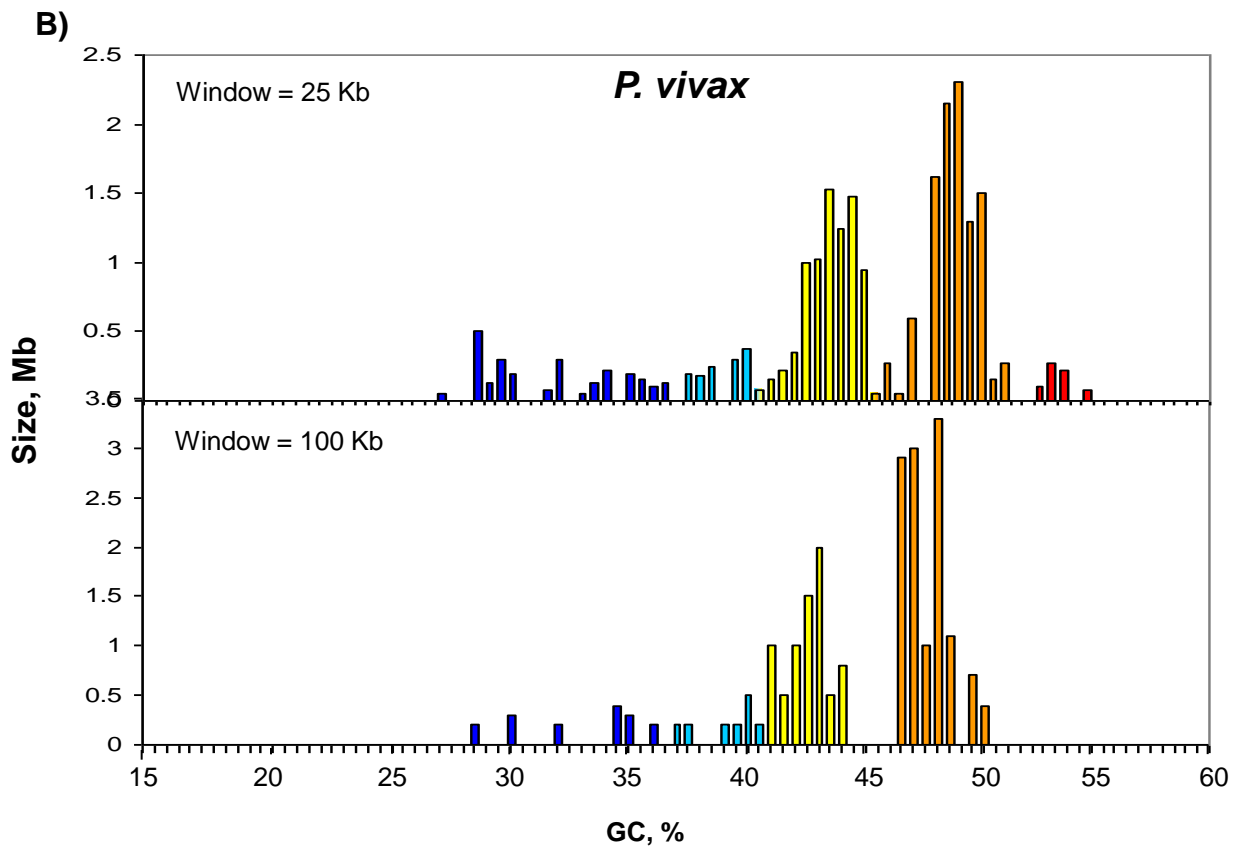

Supplement: Additional file 4: Figure S2 — Distribution by weight of DNA segments according to GC levels (A) in T. cruzi and (B) in P. vivax, considering two non-overlapping windows at 25 kb and 100 kb. [file 1471-2164-14-755-S4.pdf]
