# Supplementary material for: Compositional patterns in the genomes of unicellular eukaryotes
Source: BMC Genomics. 2013 Nov 5;14:755. doi: 10.1186/1471-2164-14-755 (PMC4007698; doi:10.1186/1471-2164-14-755)
Supplement: Additional file 3: Table S2 — Genome websites, genome size, number of chromosomes, average GC for unicellular genomes. [file 1471-2164-14-755-S3.pdf]

Additional Table T2. Genome websites, genome size, number of chromosomes, average GC for unicellular genomes.

Eukaryotes	Genome_website	Genome size, Mb	Number of chromosomes	GC, %
O. tauri	EMBL;http://www.ebi.ac.uk/embl/	12.5	20	59.2
C. merolae	NCBI; http://www.ncbi.nlm.nih.gov/	16.4	1- 6;11;12;16;18; [7-10,-13-17, 19, 20][a]	55
T. pseudonana	JGI; http://jgi.doe.gov/	30.5	24[11a-11b; 16a-16b][b]	46.9
P. tricornutum	JGI; http://jgi.doe.gov/	26.1	33	48.9
S. cerevisiae	UCSC; http://genome.ucsc.edu/	12	16	38.2
C. glabrata	NCBI; http://www.ncbi.nlm.nih.gov/	12.3	13	38.5
A. gossypii	NCBI; http://www.ncbi.nlm.nih.gov/	8.7	7	52.1
C. neoformans	NCBI; http://www.ncbi.nlm.nih.gov/	19	14	48.6
T. brucei	NCBI; http://www.ncbi.nlm.nih.gov/	21.9	10+11[c]	46.6
T. cruzi	http://tritrypdb.org/tritrypdb/	32.4	41	50.5
P. falciparum	NCBI; http://www.ncbi.nlm.nih.gov/	22	14	19.4
P. vivax	NCBI; http://www.ncbi.nlm.nih.gov/	21.6	14	45.2
P. berghei	http://protists.ensembl.org/	17.6	14	22.0
P. chabaudi	http://protists.ensembl.org/	18.1	14	22.5
P. knowlesi	http://protists.ensembl.org/	22.1	14	40.8
T. gondii	http://protists.ensembl.org/	61.8	12	52.7
D. discoideum	http://protists.ensembl.org/	33.9	6	28.4

(a) The chromosomes in square brackets are not yet assembled and are divided into scaffolds.
(b) Chromosomes 11 and 16 are divided into two scaffolds, not yet assembled.
(c) Chromosomes 11 is divided into 2 scaffolds, not yet assembled.
