# Supplementary material for: Compositional patterns in the genomes of unicellular eukaryotes
Source: BMC Genomics. 2013 Nov 5;14:755. doi: 10.1186/1471-2164-14-755 (PMC4007698; doi:10.1186/1471-2164-14-755)
Supplement: Additional file 5: Table S3 — Coordinates, sizes and GC levels of O. tauri segments. Table S4. Coordinates, sizes and GC levels of C. merolae segments. Table S5. Coordinates, sizes and GC levels of T. pseudonana segments. Table S6. Coordinates, sizes and GC levels of P. tricornutum segments. Table S7. Coordinates, sizes and GC levels of S. cerevisiae segments. Table S8. Coordinates, sizes and GC levels of C. glabrata segments. Table S9. Coordinates, sizes and GC levels of A. gossypii segments. Table S10. Coordinates, sizes and GC levels of C. neoformans segments. Table S11. Coordinates, sizes and GC levels of T. brucei segments. Table S12. Coordinates, sizes and GC levels of T. cruzi segments. Table S13. Coordinates, sizes and GC levels of P. falciparum segments. Table S14. Coordinates, sizes and GC levels of P. vivax segments. Table S15. Coordinates, sizes and GC levels of T. gondii segments. Table S16. Coordinates, sizes and GC levels of P. knowlesi segments. Table S17. Coordinates, sizes and GC levels of P. berghei segments. Table S18. Coordinates, sizes and GC levels of P. chabaudi segments. Table S19. Coordinates, sizes and GC levels of G. theta segments*. Table S20. Coordinates, sizes and GC levels of D. discoideum segments. [file 1471-2164-14-755-S5.pdf]

Additional Table T3. Coordinates, sizes and GC levels of *O. tauri* segments.

Chr	Start	End	Size (Mb)	GC, %
1	0	1.09	1.090	59.3
2	0	0.025	0.025	51.5
2	0.025	0.05	0.025	58.2
2	0.05	0.412	0.362	49.3
2	0.412	0.45	0.038	53.8
2	0.45	0.575	0.125	50.3
2	0.575	1.06	0.485	60.1
3	0	0.987	0.987	59.0
4	0	0.887	0.887	59.5
5	0	0.825	0.825	59.6
6	0	0.8	0.800	59.3
7	0	0.75	0.750	59.3
8	0	0.7	0.700	59.7
9	0	0.687	0.687	60.1
10	0	0.575	0.575	60.2
11	0	0.587	0.587	59.8
12	0	0.525	0.525	59.5
13	0	0.537	0.537	59.9
14	0	0.525	0.525	60.4
15	0	0.487	0.487	60.2
16	0	0.475	0.475	60.0
17	0	0.4	0.400	60.7
18	0	0.325	0.325	60.5
19	0	0.075	0.075	54.3
19	0.075	0.15	0.075	51.1
19	0.15	0.187	0.037	60.1
20	0	0.162	0.162	62.4

Additional Table T4. Coordinates, sizes and GC levels of *C. merolae* segments.

Chr	Start	End	Size (Mb)	GC, %
1	0	0.19	0.19	55.5
1	0.19	0.24	0.05	51.2
1	0.24	0.43	0.19	55.5
2	0	0.18	0.18	55.4
2	0.18	0.25	0.07	52.2
2	0.25	0.44	0.19	55.9
2	0.44	0.46	0.02	52.7
3	0	0.28	0.28	54.7
3	0.28	0.35	0.07	51.1
3	0.35	0.46	0.11	55.1
3	0.46	0.49	0.03	55.5
4	0	0.23	0.23	55.2
4	0.23	0.34	0.11	52.4
4	0.34	0.50	0.16	55.1
4	0.5	0.53	0.03	51.0
5	0	0.09	0.09	54.8
5	0.09	0.23	0.14	51.5
5	0.23	0.53	0.3	55.9
6	0	0.30	0.3	54.9
6	0.3	0.38	0.08	51.8
6	0.38	0.54	0.16	54.3
7.1	0	0.19	0.19	54.9
7.2	0	0.03	0.03	53.5
8.1	0	0.06	0.06	53.4
8.1	0.06	0.10	0.04	50.4
8.2	0	0.05	0.05	52.4
8.2	0.05	0.26	0.21	55.7
8.3	0	0.36	0.36	55.6
8.3	0.36	0.39	0.03	52.6
9.1	0	0.05	0.05	56.2
9.2	0	0.14	0.14	56.2
9.4	0	0.21	0.21	55.9
9.4	0.21	0.29	0.08	52.9
9.5	0	0.04	0.04	53.2
9.6	0	0.04	0.04	55.2
9.7	0	0.04	0.04	54.4
9.9	0	0.04	0.04	57.0
9.11	0	0.04	0.04	55.2
9.14	0	0.18	0.18	56.1
10.1	0	0.51	0.51	55.2
10.1	0	0.03	0.03	51.3
10.1	0.03	0.13	0.1	56.2
10.3	0	0.04	0.04	55.9
10.3	0.04	0.11	0.07	50.6
10.3	0.11	0.19	0.08	56.3
11	0	0.03	0.03	52.4
11	0.03	0.05	0.02	57.6

11	0.05	0.13	0.08	54.7
11	0.13	0.18	0.05	50.1
11	0.18	0.86	0.68	54.8
12	0	0.25	0.25	55.7
12	0.25	0.35	0.1	55.2
12	0.35	0.43	0.08	53.9
12	0.43	0.53	0.1	52.3
12	0.53	0.84	0.31	55.8
12	0.84	0.86	0.02	52.7
13.1	0	0.09	0.09	55.8
13.2	0	0.51	0.51	55.3
13.2	0.51	0.61	0.1	51.8
13.2	0.61	0.79	0.18	54.7
14.4	0	0.10	0.1	55.4
14.5	0	0.28	0.28	56.0
14.8	0	0.05	0.05	57.9
14.12	0	0.15	0.15	54.8
14.14	0	0.05	0.05	56.3
14.15	0	0.09	0.09	55.7
14.15	0.09	0.11	0.02	52.6
15.1	0	0.14	0.14	54.4
15.2	0	0.13	0.125	55.3
15.2	0.125	0.18	0.05	51.2
15.4	0	0.04	0.04	51.2
15.4	0.04	0.11	0.07	54.3
15.5	0	0.31	0.31	55.7
15.6	0	0.14	0.14	55.8
15.6	0.14	0.16	0.02	53.5
16	0	0.91	0.91	55.0
17.1	0	0.03	0.03	58.8
17.1	0.03	0.24	0.21	56.1
17.1	0.24	0.40	0.16	52.0
17.1	0.4	0.78	0.38	55.1
17.2	0	0.05	0.05	57.2
17.3	0	0.20	0.2	56.1
17.3	0.2	0.23	0.03	54.1
17.3	0.23	0.43	0.2	56.3
18	0	0.05	0.05	53.1
18	0.05	0.16	0.11	56.5
18	0.16	0.20	0.04	54.1
18	0.2	0.94	0.74	56.1
18	0.94	0.99	0.05	51.8
18	0.99	1.26	0.27	55.4
19.1	0	0.19	0.19	55.4
19.8	0	0.06	0.06	55.0
19.8	0.06	0.21	0.15	52.9
19.9	0	0.90	0.9	55.6
20.1	0	0.41	0.41	55.6
20.9	0	0.38	0.375	54.9
20.11	0	0.06	0.06	52.3

20.11	0.06	0.49	0.43	55.5
20.12	0	0.18	0.18	54.8
20.12	0.18	0.24	0.06	50.9
20.12	0.24	0.31	0.07	55.2
20.12	0.31	0.34	0.03	52.5
20.12	0.34	0.38	0.04	54.5

Additional Table T5. Coordinates, sizes and GC levels of *T. pseudonana* segments.

Chr	Start	End	Size (Mb)	GC, %
1	0	0.525	0.525	47.1
1	0.525	0.550	0.025	45.5
1	0.55	3.050	2.500	46.9
2	0	2.713	2.713	46.9
3	0	2.713	2.713	46.9
4	0	2.413	2.413	47.0
5	0	2.313	2.313	46.9
6	0	2.013	2.013	47.0
6	2.0125	2.075	0.063	45.3
7	0	2.000	2.000	47.0
8	0	1.275	1.275	46.8
9	0	1.200	1.200	47.0
10	0	0.025	0.025	44.1
10	0.025	1.088	1.063	47.2
10	1.0875	1.113	0.025	44.5
11a	0	0.175	0.175	47.1
11a	0.175	0.200	0.025	45.8
11a	0.2	0.813	0.613	46.9
11b	0	0.088	0.088	47.0
12	0	0.500	0.500	47.1
12	0.5	0.525	0.025	45.8
12	0.525	1.113	0.588	47.1
12	1.1125	1.138	0.025	44.1
13	0	1.063	1.063	47.0
14	0	1.000	1.000	47.1
15	0	0.938	0.938	47.0
16a	0	0.463	0.463	46.8
16b	0	0.150	0.150	47.2
16b	0.15	0.175	0.025	44.6
17	0	0.663	0.663	47.0
18	0	0.838	0.838	46.8
20	0	0.813	0.813	46.9
22	0	0.025	0.025	45.7
22	0.025	1.063	1.038	47.1
23	0	0.463	0.463	47.2
24	0	0.300	0.300	46.6

Additional Table T6. Coordinates, sizes and GC levels of *P. tricornutum* segments.

Chr	Start	End	Size (Mb)	GC, %
1	0	0.338	0.338	49.8
1	0.3375	0.375	0.038	45.9
1	0.375	2.538	2.163	48.3
2	0	0.600	0.600	49.0
2	0.6	0.625	0.025	44.7
2	0.625	1.500	0.875	48.4
3	0	0.963	0.963	48.8
3	0.9625	0.988	0.025	43.9
3	0.9875	1.250	0.263	48.7
3	1.25	1.275	0.025	45.7
3	1.275	1.463	0.188	48.8
4	0	0.775	0.775	48.9
4	0.775	0.800	0.025	45.0
4	0.8	1.363	0.563	48.5
5	0	0.238	0.238	49.7
5	0.2375	0.263	0.025	46.3
5	0.2625	1.100	0.838	49.1
6	0	0.025	0.025	44.3
6	0.025	1.038	1.013	49.4
7	0	0.213	0.213	49.5
7	0.2125	0.275	0.063	44.9
7	0.275	1.038	0.763	47.9
8	0	0.188	0.188	49.3
8	0.1875	0.213	0.025	42.9
8	0.2125	0.238	0.025	49.1
8	0.2375	0.263	0.025	46.8
8	0.2625	0.975	0.713	49.4
8	0.975	1.013	0.038	43.2
9	0	0.925	0.925	49.2
9	0.925	1.013	0.087	46.0
10	0	0.988	0.988	49.1
11	0	0.038	0.038	43.3
11	0.0375	0.813	0.775	49.0
11	0.8125	0.863	0.050	45.6
11	0.8625	0.925	0.063	48.4
11	0.925	0.950	0.025	45.0
12	0	0.050	0.050	46.6
12	0.05	0.163	0.113	46.6
12	0.1625	0.325	0.163	48.8
12	0.325	0.500	0.175	47.7
12	0.5	0.888	0.388	48.9
12	0.8875	0.913	0.025	44.8
13	0	0.075	0.075	45.7
13	0.075	0.888	0.813	49.0
14	0	0.263	0.263	50.1
14	0.2625	0.288	0.025	44.5
14	0.2875	0.813	0.525	49.2

14	0.8125	0.838	0.025	44.2
15	0	0.825	0.825	49.5
16	0	0.163	0.163	49.4
16	0.1625	0.188	0.025	46.0
16	0.1875	0.738	0.550	49.2
16	0.7375	0.775	0.038	44.8
17	0	0.500	0.500	49.2
17	0.5	0.525	0.025	43.9
17	0.525	0.713	0.188	49.5
18	0	0.038	0.038	45.5
18	0.0375	0.388	0.350	49.1
18	0.3875	0.413	0.025	44.6
18	0.4125	0.713	0.300	49.7
19	0	0.675	0.675	49.5
19	0.675	0.700	0.025	45.3
20	0	0.688	0.688	49.2
21	0	0.663	0.663	49.4
22	0	0.600	0.600	49.0
23	0	0.525	0.525	49.5
24	0	0.475	0.475	49.1
24	0.475	0.513	0.038	45.3
25	0	0.500	0.500	49.6
26	0	0.450	0.450	49.6
27	0	0.025	0.025	45.3
27	0.025	0.413	0.388	49.5
28	0	0.400	0.400	47.9
29	0	0.050	0.050	44.6
29	0.05	0.388	0.338	49.7
30	0	0.325	0.325	49.5
31	0	0.188	0.188	49.2
31	0.1875	0.225	0.038	45.4
31	0.225	0.263	0.038	48.1
32	0	0.163	0.163	48.2
33	0	0.100	0.100	47.2

Additional Table T7. Coordinates, sizes and GC levels of *S. cerevisiae* segments.

Chr	Start	End	Size (Mb)	GC, %
1	0.000	0.025	0.025	36.1
1	0.025	0.238	0.213	39.6
2	0.000	0.825	0.825	38.3
3	0.000	0.050	0.050	38.0
3	0.050	0.075	0.025	42.2
3	0.075	0.138	0.063	38.2
3	0.138	0.188	0.050	36.8
3	0.188	0.250	0.063	39.8
3	0.250	0.275	0.025	39.9
3	0.275	0.300	0.025	35.0
3	0.300	0.325	0.025	0.0
4	0.000	0.325	0.325	38.4
4	0.325	0.350	0.025	36.6
4	0.350	0.500	0.150	38.1
4	0.500	0.525	0.025	36.2
4	0.525	0.600	0.075	37.9
4	0.600	0.625	0.025	36.5
4	0.625	0.650	0.025	38.0
4	0.650	0.675	0.025	37.0
4	0.675	0.775	0.100	38.0
4	0.775	0.800	0.025	36.9
4	0.800	0.963	0.163	37.9
4	0.963	0.988	0.025	37.2
4	0.988	1.013	0.025	37.2
4	1.013	1.038	0.025	37.9
4	1.038	1.100	0.063	37.2
4	1.100	1.125	0.025	35.7
4	1.125	1.200	0.075	37.9
4	1.200	1.225	0.025	36.0
4	1.225	1.538	0.313	38.2
5	0.000	0.588	0.588	38.5
6	0.000	0.125	0.125	39.2
6	0.125	0.163	0.038	36.0
6	0.163	0.275	0.113	39.0
7	0.000	1.100	1.100	38.0
8	0.000	0.388	0.388	38.5
8	0.388	0.550	0.163	38.2
8	0.550	0.575	0.025	0.0
9	0.000	0.375	0.375	38.7
9	0.375	0.400	0.025	41.5
9	0.400	0.450	0.050	40.1
10	0.000	0.750	0.750	38.4
11	0.000	0.025	0.025	36.6
11	0.025	0.438	0.413	38.2
11	0.438	0.463	0.025	36.9
11	0.463	0.513	0.050	37.6
11	0.513	0.538	0.025	39.8

11	0.538	0.675	0.138	38.1
12	0.000	0.138	0.138	39.2
12	0.138	0.163	0.025	37.0
12	0.163	0.450	0.288	38.3
12	0.450	0.475	0.025	43.0
12	0.475	0.663	0.188	38.9
12	0.663	0.700	0.038	36.6
12	0.700	0.738	0.038	37.8
12	0.738	0.763	0.025	37.9
12	0.763	0.963	0.200	38.3
12	0.963	0.988	0.025	37.5
12	0.988	1.038	0.050	38.2
12	1.038	1.063	0.025	36.7
12	1.063	1.088	0.025	39.3
13	0.000	0.513	0.513	38.3
13	0.513	0.538	0.025	37.1
13	0.538	0.925	0.388	38.1
14	0.000	0.788	0.788	38.6
15	0.000	0.350	0.350	38.3
15	0.350	0.375	0.025	36.3
15	0.375	1.100	0.725	38.1
16	0.000	0.763	0.763	38.1
16	0.763	0.788	0.025	37.2
16	0.788	0.950	0.163	38.2

Additional Table T8. Coordinates, sizes and GC levels of *C. glabrata* segments.

Chr	Start	End	Size (Mb)	GC, %
A	0	0.100	0.100	37.9
A	0.1	0.150	0.050	43.8
A	0.15	0.300	0.150	39.7
A	0.3	0.325	0.025	42.3
A	0.325	0.388	0.063	38.4
A	0.3875	0.450	0.063	42.6
A	0.45	0.488	0.038	35.6
B	0	0.075	0.075	35.7
B	0.075	0.138	0.063	40.2
B	0.1375	0.175	0.038	35.9
B	0.175	0.225	0.050	38.5
B	0.225	0.250	0.025	44.0
B	0.25	0.275	0.025	37.7
B	0.275	0.300	0.025	41.5
B	0.3	0.513	0.213	39.4
C	0	0.088	0.088	38.0
C	0.0875	0.113	0.025	44.5
C	0.1125	0.163	0.050	38.9
C	0.1625	0.213	0.050	42.2
C	0.2125	0.338	0.125	39.6
C	0.3375	0.363	0.025	44.7
C	0.3625	0.425	0.063	38.1
C	0.425	0.450	0.025	45.4
C	0.45	0.538	0.088	39.0
C	0.5375	0.563	0.025	33.0
D	0	0.163	0.163	38.3
D	0.1625	0.225	0.063	39.3
D	0.225	0.288	0.063	36.2
D	0.2875	0.388	0.100	40.2
D	0.3875	0.413	0.025	42.8
D	0.4125	0.663	0.250	39.5
E	0	0.050	0.050	38.2
E	0.05	0.175	0.125	41.9
E	0.175	0.625	0.450	38.7
E	0.625	0.688	0.063	36.6
F	0	0.038	0.038	35.0
F	0.0375	0.200	0.163	39.2
F	0.2	0.250	0.050	36.5
F	0.25	0.388	0.138	38.9
F	0.3875	0.413	0.025	34.7
F	0.4125	0.500	0.088	38.5
F	0.5	0.613	0.113	36.8
F	0.6125	0.788	0.175	38.8
F	0.7875	0.813	0.025	42.6
F	0.8125	0.888	0.075	38.4
F	0.8875	0.938	0.050	34.0
G	0	0.163	0.163	38.8

G	0.1625	0.188	0.025	45.2
G	0.1875	0.275	0.088	38.1
G	0.275	0.325	0.050	36.4
G	0.325	0.538	0.213	38.6
G	0.5375	0.563	0.025	36.6
G	0.5625	0.688	0.125	38.8
G	0.6875	0.713	0.025	43.0
G	0.7125	0.750	0.038	37.3
G	0.75	0.963	0.213	38.7
G	0.9625	1.000	0.038	33.4
H	0	0.050	0.050	35.5
H	0.05	0.100	0.050	39.9
H	0.1	0.313	0.213	37.7
H	0.3125	0.400	0.088	39.1
H	0.4	0.475	0.075	37.6
H	0.475	0.725	0.250	37.9
H	0.725	0.800	0.075	36.2
H	0.8	1.013	0.213	39.4
H	1.0125	1.063	0.050	36.5
I	0	0.125	0.125	39.0
I	0.125	0.400	0.275	38.6
I	0.4	0.425	0.025	35.6
I	0.425	0.525	0.100	37.0
I	0.525	0.638	0.113	38.8
I	0.6375	0.663	0.025	36.1
I	0.6625	0.763	0.100	39.7
I	0.7625	0.800	0.038	35.2
I	0.8	1.038	0.238	39.9
I	1.0375	1.100	0.063	34.4
J	0	0.088	0.088	37.7
J	0.0875	0.125	0.038	46.1
J	0.125	0.200	0.075	40.7
J	0.2	0.225	0.025	35.7
J	0.225	0.275	0.050	39.0
J	0.275	0.300	0.025	45.7
J	0.3	0.400	0.100	40.9
J	0.4	0.550	0.150	37.7
J	0.55	0.588	0.038	41.2
J	0.5875	0.725	0.138	36.3
J	0.725	0.750	0.025	39.1
J	0.75	0.800	0.050	35.8
J	0.8	1.200	0.400	38.7
K	0	0.150	0.150	38.4
K	0.15	0.175	0.025	44.1
K	0.175	0.250	0.075	39.5
K	0.25	0.275	0.025	35.1
K	0.275	0.438	0.163	38.2
K	0.4375	0.463	0.025	35.5
K	0.4625	0.588	0.125	38.5
K	0.5875	0.675	0.088	38.1

K	0.675	1.213	0.538	38.4
K	1.2125	1.238	0.025	34.3
K	1.2375	1.263	0.025	37.3
K	1.2625	1.313	0.050	35.2
L	0	0.025	0.025	44.0
L	0.025	0.075	0.050	35.7
L	0.075	0.125	0.050	39.5
L	0.125	0.188	0.063	37.7
L	0.1875	0.238	0.050	36.0
L	0.2375	0.388	0.150	38.6
L	0.3875	0.638	0.250	38.1
L	0.6375	0.675	0.038	34.9
L	0.675	0.963	0.288	38.0
L	0.9625	1.063	0.100	37.4
L	1.0625	1.088	0.025	41.7
L	1.0875	1.300	0.213	39.3
L	1.3	1.325	0.025	35.7
L	1.325	1.388	0.063	42.4
L	1.3875	1.450	0.063	37.9
M	0	0.063	0.063	35.3
M	0.0625	0.100	0.038	39.4
M	0.1	0.388	0.288	38.3
M	0.3875	1.138	0.750	38.9
M	1.1375	1.163	0.025	36.8
M	1.1625	1.188	0.025	41.6
M	1.1875	1.313	0.125	39.3
M	1.3125	1.413	0.100	37.0

Additional Table T9. Coordinates, sizes and GC levels of *A. gossypii* segments.

Chr	Start	End	Size (Mb)	GC, %
1	0	0.2	0.2	48.5
1	0.2	0.7	0.5	53.6
2	0	0.3	0.3	50.0
2	0.3	0.6	0.3	53.9
2	0.6	0.9	0.3	50.2
3	0	0.5	0.5	52.4
3	0.5	0.8	0.3	54.7
3	0.8	1.0	0.2	51.7
4	0	0.4	0.4	51.5
4	0.4	0.6	0.2	54.2
4	0.6	0.8	0.2	51.7
4	0.8	1.2	0.4	53.2
4	1.2	1.5	0.3	50.8
5	0	0.2	0.2	51.1
6	0	0.2	0.2	51.7
7	0	0.1	0.1	48.9
7	0.1	0.3	0.2	54.1
7	0.3	0.4	0.1	52.0
7	0.4	0.5	0.1	48.4
7	0.5	0.7	0.2	53.3
7	0.7	0.8	0.1	50.4
7	0.8	0.9	0.1	51.1
7	0.9	1.1	0.2	51.5
7	1.1	1.3	0.2	53.6
7	1.3	1.4	0.1	50.8
7	1.4	1.5	0.1	53.5

Additional Table T10. Coordinates, sizes and GC levels of *C. neoformans* segments.

Chr	Start	End	Size (Mb)	GC, %
1	0	0.45	0.450	48.9
1	0.45	0.65	0.200	48.4
1	0.65	0.95	0.300	47.7
1	0.95	0.99	0.040	50.6
1	0.99	1.59	0.600	48.2
1	1.59	1.61	0.020	52.7
1	1.61	2.31	0.700	49.0
2	0	0.8375	0.838	48.4
2	0.8375	0.9	0.063	49.3
2	0.9	1.64	0.740	48.3
3	0	0.7	0.700	48.2
3	0.7	0.825	0.125	47.1
3	0.825	0.9	0.075	51.4
3	0.9	0.925	0.025	46.1
3	0.925	0.975	0.050	50.3
3	0.975	1.2125	0.238	48.1
3	1.2125	1.2375	0.025	45.4
3	1.2375	1.4625	0.225	48.5
3	1.4625	2.1125	0.650	48.6
4	0	0.2125	0.213	48.1
4	0.2125	0.2625	0.050	50.0
4	0.2625	0.2875	0.025	45.2
4	0.2875	0.8375	0.550	48.7
4	0.8375	0.8625	0.025	46.7
4	0.8625	1.05	0.188	48.8
4	1.05	1.2625	0.213	48.2
4	1.2625	1.525	0.263	49.2
4	1.525	1.5875	0.063	46.1
4	1.5875	1.6125	0.025	49.1
4	1.6125	1.7875	0.175	48.4
5	0	0.0875	0.088	49.4
5	0.0875	0.3875	0.300	48.6
5	0.3875	0.75	0.363	48.4
5	0.75	0.775	0.025	45.2
5	0.775	0.85	0.075	50.2
5	0.85	1.5125	0.663	48.9
6	0	0.4	0.400	48.5
6	0.4	0.4375	0.038	46.0
6	0.4375	0.55	0.113	49.1
6	0.55	0.775	0.225	49.0
6	0.775	0.875	0.100	45.7
6	0.875	0.925	0.050	50.7
6	0.925	1.4	0.475	48.3
6	1.4	1.45	0.050	49.9
7	0	0.725	0.725	48.5
7	0.725	0.75	0.025	52.9
7	0.75	0.875	0.125	48.1

7	0.875	0.9125	0.038	45.4
7	0.9125	1.2125	0.300	48.8
7	1.2125	1.35	0.138	48.4
8	0	0.75	0.750	48.7
8	0.75	1.2	0.450	48.3
9	0	0.375	0.375	48.9
9	0.375	0.4125	0.038	45.9
9	0.4125	0.4375	0.025	50.3
9	0.4375	0.4625	0.025	47.0
9	0.4625	0.8125	0.350	48.3
9	0.8125	0.8375	0.025	49.8
9	0.8375	1.1875	0.350	48.5
10	0	0.5375	0.538	48.9
10	0.5375	0.5625	0.025	51.5
10	0.5625	1.0875	0.525	48.6
11	0	1.025	1.025	48.6
12	0	0.1625	0.163	48.8
12	0.1625	0.1875	0.025	44.6
12	0.1875	0.9125	0.725	48.4
13	0	0.1375	0.138	48.6
13	0.1375	0.175	0.038	51.9
13	0.175	0.425	0.250	48.0
13	0.425	0.45	0.025	50.4
13	0.45	0.6125	0.163	49.7
13	0.6125	0.8	0.188	47.5
14	0	0.775	0.775	48.7

Additional Table T11. Coordinates, sizes and GC levels of *T. brucei* segments.

Chr	Start	End	Size (Mb)	GC, %
I	0.0	0.200	0.20	41.2
I	0.200	1.080	0.88	46.2
II	0.000	0.250	0.25	39.5
II	0.250	1.100	0.85	46.4
II	1.100	1.200	0.10	39.1
III	0.0	0.100	0.10	40.4
III	0.100	1.575	1.48	47.2
III	1.575	1.675	0.10	43.1
IV	0.0	0.075	0.08	39.2
IV	0.075	0.500	0.43	47.3
IV	0.500	1.500	1.00	47.3
IV	1.500	1.600	0.10	40.8
V	0.0	1.375	1.38	46.8
V	1.375	1.625	0.25	41.1
VI	0.0	0.050	0.05	41.6
VI	0.050	0.150	0.10	45.6
VII	0.000	0.475	0.48	47.0
VII	0.475	0.575	0.10	39.6
VII	0.575	2.225	1.65	47.4
VIII	0.0	0.300	0.30	42.9
VIII	0.300	1.000	0.70	49.2
VIII	1.000	1.175	0.18	46.6
VIII	1.175	2.500	1.33	47.6
IX	0.0	0.100	0.10	42.3
IX	0.100	0.150	0.05	39.9
X	0.0	0.225	0.23	44.8
X	0.225	3.925	3.70	48.1
X	3.925	4.075	0.15	40.1
XI	0.0	4.500	4.50	47.8
XI	4.500	5.275	0.78	41.8
XI	0.0	0.125	0.13	43.0
XI	0.125	0.300	0.18	41.4

Additional Table T12. Coordinates, sizes and GC levels of *T. cruzi* segments.

Chr	Start	End	Size (Mb)	GC, %
1	0.000	0.080	0.080	51.9
2	0.000	0.050	0.050	54.3
2	0.050	0.100	0.050	49.3
3	0.000	0.100	0.100	47.3
3	0.100	0.150	0.050	53.5
3	0.150	0.200	0.050	48.9
4	0.000	0.150	0.150	49.2
4	0.150	0.200	0.050	53.6
5	0.000	0.125	0.125	53.6
5	0.125	0.230	0.105	49.2
6	0.000	0.200	0.200	49.0
6	0.200	0.300	0.100	53.0
6	0.300	0.400	0.100	50.5
7	0.000	0.375	0.375	50.6
8	0.000	0.399	0.399	49.7
9	0.000	0.510	0.510	49.1
10	0.000	0.225	0.225	49.2
10	0.225	0.275	0.050	54.0
10	0.275	0.520	0.245	47.6
11	0.000	0.075	0.075	52.4
11	0.075	0.125	0.050	57.5
11	0.125	0.540	0.415	49.4
12	0.000	0.050	0.050	52.4
12	0.050	0.100	0.050	55.7
12	0.100	0.500	0.400	49.4
13	0.000	0.325	0.325	48.0
13	0.325	1.075	0.750	52.4
13	1.075	1.242	0.167	50.7
14	0.000	0.200	0.200	52.2
14	0.200	0.250	0.050	55.4
14	0.250	0.600	0.350	48.0
15	0.000	0.125	0.125	48.6
15	0.125	0.275	0.150	54.2
15	0.275	0.425	0.150	50.0
16	0.000	0.075	0.075	56.0
16	0.075	0.475	0.400	49.4
16	0.475	0.525	0.050	53.0
16	0.525	0.665	0.140	52.5
17	0.000	0.175	0.175	53.8
17	0.175	0.475	0.300	49.0
17	0.475	0.667	0.192	52.0
18	0.000	0.250	0.250	52.7
18	0.250	0.677	0.427	53.8
19	0.000	0.125	0.125	54.0
19	0.125	0.500	0.375	48.0
19	0.500	0.657	0.157	56.2
20	0.000	0.475	0.475	48.4

20	0.475	0.672	0.197	53.8
21	0.000	0.250	0.250	50.7
21	0.250	0.325	0.075	45.1
21	0.325	0.715	0.390	49.2
22	0.000	0.250	0.250	53.0
22	0.250	0.720	0.470	48.9
23	0.000	0.175	0.175	50.0
23	0.175	0.225	0.050	54.3
23	0.225	0.550	0.325	49.6
23	0.550	0.650	0.100	55.0
24	0.000	0.275	0.275	55.2
24	0.275	0.475	0.200	50.3
24	0.475	0.775	0.300	53.2
24	0.775	0.809	0.034	48.2
25	0.000	0.100	0.100	55.7
25	0.100	0.400	0.300	50.4
25	0.400	0.475	0.075	54.0
25	0.475	0.675	0.200	51.3
25	0.675	0.840	0.165	56.0
26	0.000	0.075	0.075	55.0
26	0.075	0.225	0.150	49.0
26	0.225	0.350	0.125	52.1
26	0.350	0.750	0.400	50.0
26	0.750	0.816	0.066	54.3
27	0.000	0.800	0.800	48.8
28	0.000	0.075	0.075	53.5
28	0.075	0.250	0.175	50.4
28	0.250	0.650	0.400	55.4
28	0.650	0.725	0.075	51.1
29	0.000	0.450	0.450	54.5
29	0.450	0.874	0.424	47.7
30	0.000	0.775	0.775	49.9
30	0.775	0.850	0.075	55.0
31	0.000	0.300	0.300	53.6
31	0.300	0.775	0.475	51.5
31	0.475	0.525	0.050	56.4
31	0.525	0.960	0.435	49.6
32	0.125	0.225	0.100	50.1
32	0.300	0.969	0.669	47.7
33	0.000	0.225	0.225	50.0
33	0.225	0.525	0.300	55.4
33	0.525	0.775	0.250	49.8
33	0.775	0.825	0.050	55.7
33	0.825	1.053	0.228	51.8
34	0.000	0.001	0.001	53.0
35	0.000	1.115	1.115	49.7
35	1.115	1.680	0.565	54.5
36	0.000	1.190	1.190	48.9
37	0.000	2.480	2.480	48.3
38	0.000	0.275	0.275	49.0

38	0.275	0.325	0.050	53.8
38	0.325	0.525	0.200	51.5
38	0.530	0.600	0.070	56.5
38	0.600	0.966	0.366	51.9
39	0.000	1.860	1.860	47.8
40	0.000	0.050	0.050	53.2
40	0.050	0.100	0.050	51.0
40	0.100	1.075	0.975	47.8
40	1.075	1.675	0.600	54.0
40	1.675	1.950	0.275	48.6
41	0.000	0.050	0.050	36.3
41	0.050	0.150	0.100	50.0
41	0.150	0.325	0.175	51.2
41	0.325	0.525	0.200	52.4
41	0.525	0.675	0.150	51.0
41	0.675	0.850	0.175	54.0
41	1.000	1.075	0.075	50.7
41	1.075	2.250	1.175	53.4
41	2.250	2.425	0.175	48.8

Additional Table T13. Coordinates, sizes and GC levels of *P. falciparum* segments.

Chr	Start	End	Size (Mb)	GC, %
1	0	0.050	0.05	29.0
1	0.050	0.475	0.43	18.8
1	0.475	0.600	0.13	19.9
1	0.600	0.650	0.05	27.1
2	0	0.050	0.05	27.2
2	0.050	0.900	0.85	18.9
2	0.900	0.950	0.05	23.8
3	0	0.050	0.05	29.9
3	0.050	1.025	0.98	19.0
3	1.025	1.075	0.05	29.9
4	0	0.075	0.08	30.0
4	0.075	0.550	0.48	19.1
4	0.550	0.625	0.08	24.7
4	0.625	0.925	0.30	18.4
4	0.925	1.000	0.08	24.8
4	1.000	1.150	0.15	18.5
4	1.150	1.225	0.08	28.0
5	0	1.350	1.35	19.2
6	0	0.125	0.13	25.8
6	0.125	1.150	1.03	18.8
6	1.150	1.200	0.05	22.4
6	1.200	1.350	0.15	18.7
6	1.350	1.400	0.05	25.0
7	0	0.025	0.03	32.2
7	0.025	0.400	0.38	18.8
7	0.400	0.500	0.10	24.7
7	0.500	1.325	0.83	19.2
7	1.325	1.375	0.05	30.3
8	0	0.025	0.03	30.8
8	0.025	0.875	0.85	18.5
8	0.0875	0.925	0.84	18.7
8	0.925	1.3	0.38	20.4

Additional Table T14. Coordinates, sizes and GC levels of *P. vivax* segments.

Chr	Start	End	Size (Mb)	GC, %
1	0	0.05	0.05	36.3
1	0.05	0.275	0.225	45
1	0.275	0.45	0.175	50.3
1	0.45	0.525	0.075	53.8
1	0.525	0.6	0.075	44.9
1	0.6	0.725	0.125	53.8
1	0.725	0.775	0.05	47.1
1	0.775	0.83	0.055	33.7
2	0	0.05	0.05	27.3
2	0.05	0.1	0.05	38.1
2	0.1	0.2	0.1	42.6
2	0.2	0.35	0.15	53.2
2	0.35	0.55	0.2	49.6
2	0.55	0.675	0.125	45
2	0.675	0.755	0.08	35
3	0	0.05	0.05	39.9
3	0.05	0.375	0.325	48.1
3	0.375	0.5	0.125	52
3	0.5	0.575	0.075	41.3
3	0.575	0.65	0.075	54.5
3	0.65	0.725	0.075	53.3
3	0.725	0.8	0.075	48.5
3	0.8	0.85	0.05	35.8
3	0.85	1.01	0.16	26.8
4	0	0.05	0.05	26.4
4	0.05	0.1	0.05	35.5
4	0.1	0.3	0.2	50.1
4	0.3	0.45	0.15	39.3
4	0.45	0.725	0.275	49.8
4	0.725	0.775	0.05	45.5
4	0.775	0.876	0.101	34
5	0	0.05	0.05	27.4
5	0.05	0.125	0.075	35.4
5	0.125	0.175	0.05	44.6
5	0.175	0.525	0.35	48.6
5	0.525	0.575	0.05	45.1
5	0.575	0.725	0.15	50.4
5	0.725	0.825	0.1	43.4
5	0.825	0.925	0.1	35.9
5	0.925	1.225	0.3	48.9
5	1.225	1.275	0.05	43.8
5	1.275	1.371	0.096	29.3
6	0	0.05	0.05	33.5
6	0.05	0.15	0.1	50.1
6	0.15	0.25	0.1	53.9
6	0.25	0.325	0.075	49.1
6	0.325	0.425	0.1	27.6

6	0.425	0.55	0.125	42.4
6	0.55	1	0.45	49.2
6	1	1.033	0.033	42.5
7	0	0.075	0.075	37.7
7	0.075	0.25	0.175	42.3
7	0.25	0.625	0.375	47.1
7	0.625	0.7	0.075	46
7	0.7	1.15	0.45	49.6
7	1.15	1.225	0.075	41.8
7	1.225	1.325	0.1	53.2
7	1.325	1.4	0.075	44.7
7	1.4	1.498	0.098	29.6
8	0	0.15	0.15	34.2
8	0.15	0.4	0.25	44.9
8	0.4	0.45	0.05	39.6
8	0.45	0.725	0.275	43.4
8	0.725	1.625	0.9	49.2
8	1.625	1.678	0.053	30.8
9	0	0.05	0.05	36.9
9	0.05	0.1	0.05	45.8
9	0.1	0.375	0.275	47.4
9	0.375	0.45	0.075	44.6
9	0.45	0.725	0.275	49.8
9	0.725	0.775	0.05	36
9	0.775	1.2	0.425	49
9	1.2	1.875	0.675	44.7
9	1.875	1.923	0.048	35.4
10	0	0.075	0.075	36.8
10	0.075	0.525	0.45	43.5
10	0.525	0.875	0.35	50.3
10	0.875	0.925	0.05	38.8
10	0.925	1.225	0.3	50.8
10	1.225	1.35	0.125	37.9
10	1.35	1.42	0.07	27.9
11	0	0.075	0.075	32.8
11	0.075	0.825	0.75	44.3
11	0.825	0.9	0.075	40.8
11	0.9	1.125	0.225	44.2
11	1.125	1.55	0.425	49.7
11	1.55	1.6	0.05	38.9
11	1.6	1.95	0.35	50.1
11	1.95	2.067	0.117	32.6
12	0	0.05	0.05	33.2
12	0.05	0.125	0.075	43.9
12	0.125	0.3	0.175	46.1
12	0.3	0.575	0.275	44.8
12	0.575	0.7	0.125	46.7
12	0.7	0.775	0.075	44.4
12	0.775	1	0.225	49.8
12	1	1.05	0.05	38.7

12	1.05	1.375	0.325	50.5
12	1.375	1.5	0.125	44.7
12	1.5	1.575	0.075	47.1
12	1.575	1.65	0.075	43
12	1.65	1.75	0.1	39.9
12	1.75	2.375	0.625	43.6
12	2.375	2.425	0.05	48.5
12	2.425	2.575	0.15	43.3
12	2.575	2.625	0.05	40.4
12	2.625	2.875	0.25	43.3
12	2.875	3.005	0.13	35.6
13	0	0.05	0.05	36.7
13	0.05	0.1	0.05	42.3
13	0.1	0.225	0.125	47.1
13	0.225	0.425	0.2	44.2
13	0.425	1.25	0.825	48.5
13	1.25	2	0.75	43.9
13	2	2.032	0.032	33.9
14	0	0.1	0.1	27.8
14	0.1	0.15	0.05	33.7
14	0.15	0.2	0.05	39.2
14	0.2	0.65	0.45	42.4
14	0.65	0.7	0.05	46.6
14	0.7	1.25	0.55	42.6
14	1.25	1.4	0.15	40
14	1.4	1.475	0.075	42.2
14	1.475	1.675	0.2	40.1
14	1.675	1.775	0.1	43.9
14	1.775	2.025	0.25	50.1
14	2.025	2.1	0.075	53.5
14	2.1	2.175	0.075	43.1
14	2.175	2.45	0.275	50.8
14	2.45	2.675	0.225	45.4
14	2.675	2.875	0.2	48.1
14	2.875	2.925	0.05	44.1
14	2.925	3.12	0.195	28.7

Additional Table T15. Coordinates, sizes and GC levels of *T. gondii* segments.

Chr	Start	End	Size, Mb	GC,%
Ia	0	0.05	0.05	50.1
Ia	0.05	0.125	0.075	52.9
Ia	0.125	0.3	0.175	54.9
Ia	0.3	1.075	0.775	51.2
Ia	1.075	1.35	0.275	53.1
Ia	1.35	1.400	0.05	54.3
Ia	1.400	1.575	0.175	52.4
Ia	1.575	1.700	0.125	55.6
Ia	1.700	1.899	0.199	53.8
Ib	0	0.5	0.5	51.2
Ib	0.5	0.6	0.1	55
Ib	0.6	0.925	0.325	58.9
Ib	0.925	0.975	0.05	56.6
Ib	0.975	1.575	0.6	52.3
Ib	1.575	1.625	0.05	54.5
Ib	1.625	1.75	0.125	52.2
Ib	1.75	1.85	0.1	53.7
Ib	1.85	1.925	0.075	51
Ib	1.925	1.956	0.031	55.2
II	0	0.25	0.25	52
II	0.25	0.3	0.05	53.2
II	0.3	0.55	0.25	51.7
II	0.55	0.6	0.05	54.3
II	0.6	0.95	0.35	51
II	0.95	1.025	0.075	54.4
II	1.025	1.65	0.625	51.1
II	1.65	1.7	0.05	54.1
II	1.7	1.9	0.2	50.7
II	1.9	1.975	0.075	55.2
II	1.975	2.25	0.275	51
II	2.25	2	0.053	54.6
III	0	0.1	0.1	54.3
III	0.1	0.5	0.4	52.1
III	0.5	0.575	0.075	54
III	0.575	0.625	0.05	51.2
III	0.625	0.700	0.075	54.2
III	0.700	1.125	0.425	52.1
III	1.125	1.275	0.15	55.3
III	1.275	2.025	0.75	52.1

III	2.025	2.2	0.175	54.5
III	2.2	2.471	0.271	52
IV	0	0.05	0.05	54.8
IV	0.05	0.275	0.225	51.5
IV	0.275	0.325	0.05	53.5
IV	0.325	0.475	0.15	51.6
IV	0.475	0.525	0.05	53.9
IV	0.525	1.275	0.75	52
IV	1.275	1.65	0.375	53.8
IV	1.65	2.025	0.375	51.9
IV	2.025	2.175	0.15	53.6
IV	2.175	2.3	0.125	50.7
IV	2.3	2.5	0.2	53.2
IV	2.5	2.596	0.096	51.9
V	0	0.05	0.05	54.3
V	0.05	0.225	0.175	52.1
V	0.225	0.375	0.15	53.8
V	0.375	0.550	0.175	52.1
V	0.550	0.675	0.125	54.4
V	0.675	0.750	0.075	51
V	0.750	0.975	0.225	54.8
V	0.975	1.33	0.355	50.5
V	1.33	1.40	0.07	53.9
V	1.40	3.156	1.756	52
VI	0	0.35	0.35	51.8
VI	0.35	0.425	0.075	53.8
VI	0.425	0.6	0.175	52.3
VI	0.6	0.650	0.05	56.8
VI	0.650	1.025	0.375	50.7
VI	1.025	1.075	0.05	55.4
VI	1.075	1.150	0.075	51.0
VI	1.150	1.225	0.075	54.2
VI	1.225	1.500	0.275	50.7
VI	1.500	1.600	0.1	52.8
VI	1.600	1.700	0.1	52
VI	2	1.75	0.05	54
VI	1.75	2.075	0.325	51.7
VI	2.075	2.125	0.05	55
VI	2.125	2.575	0.45	51.7
VI	2.575	2.625	0.05	54.7
VI	2.625	3.250	0.625	51.4
VI	3.250	3.350	0.1	53.2
VI	3.350	3.575	0.225	51.7

VI	3.575	3.600	0.025	53.8
VIIa	0	0.075	0.075	53.5
VIIa	0.075	0.15	0.075	51
VIIa	0.15	0.3	0.15	54.1
VIIa	0.3	0.525	0.225	53.2
VIIa	0.525	0.8	0.275	54.1
VIIa	0.8	0.925	0.125	51.2
VIIa	0.925	1.050	0.125	51.7
VIIa	1.050	1.125	0.075	53.9
VIIa	1.125	1.200	0.075	51.3
VIIa	1.200	1.325	0.125	54.7
VIIa	1.325	1.5	0.175	50.9
VIIa	1.5	1.6	0.1	55.2
VIIa	1.6	1.715	0.115	51.2
VIIa	1.715	1.825	0.11	54.4
VIIa	1.825	2.025	0.2	53.7
VIIa	2.025	2.075	0.05	55.5
VIIa	2.075	2.125	0.05	50.5
VIIa	2.125	2.175	0.05	53.6
VIIa	2.175	2.525	0.35	52.5
VIIa	2.525	2.575	0.05	54.3
VIIa	2.575	3.9	1.325	52
VIIa	3.9	4.1	0.2	54.7
VIIa	4.1	4.45	0.35	52.2
VIIa	4.45	4.50	0.05	55.1
VIIb	0	0.1	0.1	52.3
VIIb	0.1	0.150	0.05	53.9
VIIb	0.150	2.625	2.475	52
VIIb	2.625	2.675	0.05	54.3
VIIb	2.675	2.9	0.225	51.6
VIIb	2.9	2.95	0.05	54.3
VIIb	2.95	3.75	0.8	51.6
VIIb	3.75	3.80	0.05	54.5
VIIb	3.80	3.875	0.075	52.3
VIIb	3.875	4.025	0.15	54
VIIb	4.025	5.024	0.999	52
VIII	0	0.075	0.075	51.1
VIII	0.075	0.225	0.15	53.7
VIII	0.225	0.325	0.1	50.7
VIII	0.325	0.450	0.125	55.2
VIII	0.450	0.500	0.05	50.7
VIII	0.500	0.55	0.05	57.4
VIII	0.55	1.8	1.25	51.8

VIII	1.8	1.85	0.05	57.1
VIII	1.85	1.9	0.05	52.4
VIII	1.9	1.975	0.075	54.6
VIII	1.975	2.425	0.45	52.1
VIII	2.425	2.475	0.05	56.5
VIII	2.475	3.075	0.6	52
VIII	3.075	3.125	0.05	57.2
VIII	3.125	3.225	0.1	52.5
VIII	3.225	3.275	0.05	54.3
VIII	3.275	3.325	0.05	50.9
VIII	3.325	3.375	0.05	53.8
VIII	3.375	3.5	0.125	50.3
VIII	3.5	3.55	0.05	53.9
VIII	3.55	5	1.45	52.1
VIII	5	5.05	0.05	55.5
VIII	5.05	5.6	0.55	52.3
VIII	5.6	5.7	0.1	54.5
VIII	5.7	5.975	0.275	52.1
VIII	5.975	6.1	0.125	54.8
VIII	6.1	6.275	0.175	51.5
VIII	6.275	6.325	0.05	56.9
VIII	6.325	6.45	0.125	51.5
VIII	6.45	6.75	0.3	55.8
VIII	6.75	6.92	0.17	51.4
IX	0	0.1	0.1	51.5
IX	0.1	0.15	0.05	56.6
IX	0.15	1.25	1.1	51.9
IX	1.25	1.3	0.05	53.8
IX	1.3	1.45	0.15	51
IX	1.45	1.65	0.2	54.6
IX	1.65	1.725	0.075	50.8
IX	1.725	1.775	0.05	54.2
IX	1.775	1.975	0.2	51.5
IX	1.975	2.225	0.25	53.8
IX	2.225	2.325	0.1	52.4
IX	2.325	2.4	0.075	53.8
IX	2.4	2.5	0.1	52.4
IX	2.5	2.55	0.05	54.1
IX	2.55	2.875	0.325	52.3
IX	2.875	2.95	0.075	55.3
IX	2.95	3.35	0.4	51.5
IX	3.35	3.4	0.05	55.7
IX	3.4	4.575	1.175	51.8

IX	4.575	4.875	0.3	54.3
IX	4.875	4.95	0.075	52.1
IX	4.95	5	0.05	55.3
IX	5	6.4	1.4	52.3
X	0	0.1	0.1	51.2
X	0.1	0.175	0.075	55.8
X	0.175	0.575	0.4	53
X	0.575	0.650	0.075	54.1
X	0.650	1.25	0.6	52.7
X	1.25	1.325	0.075	56.7
X	1.325	1.375	0.05	52.7
X	1.375	1.45	0.075	53.2
X	1.45	2.525	1.075	52.7
X	2.525	2.575	0.05	55.3
X	2.575	3.025	0.45	52.6
X	3.025	3.175	0.15	55.4
X	3.175	4.775	1.6	52.4
X	4.775	4.825	0.05	55.2
X	4.825	5.4	0.575	51.2
X	5.4	5.45	0.05	54.3
X	5.45	5.75	0.3	52.4
X	5.75	5.975	0.225	53.1
X	5.975	6.025	0.05	55.3
X	6.025	6.475	0.45	52.5
X	6.475	6.725	0.25	54.6
X	6.725	6.95	0.225	50.8
X	6.95	7.000	0.05	56
X	7	7.419	0.419	52.3
XI	0	0.475	0.475	52.3
XI	0.475	0.550	0.075	55.9
XI	0.550	1.075	0.525	52.3
XI	1.075	1.775	0.7	55.1
XI	1.775	1.850	0.075	52.7
XI	1.850	1.925	0.075	54.8
XI	1.925	3.525	1.6	52.5
XI	3.525	3.575	0.05	58
XI	3.575	4.325	0.75	51.7
XI	4.325	4.4	0.075	55.1
XI	4.4	6.3	1.9	52
XI	6.3	6.35	0.05	56.5
XI	6.35	6.622	0.272	53
XII	0	0.70	0.7	52
XII	0.70	0.75	0.05	55.1

XII	0.75	0.875	0.125	51.5
XII	0.875	0.925	0.05	55.9
XII	0.925	2.675	1.75	52
XII	2.675	2.7	0.025	55.6
XII	2.7	3.475	0.775	52.4
XII	3.475	3.525	0.05	55.7
XII	3.525	3.875	0.35	51.3
XII	3.875	3.925	0.05	57.2
XII	3.925	5.15	1.225	51.9
XII	5.15	5.225	0.075	55.3
XII	5.225	5.425	0.2	53.3
XII	5.425	5.475	0.05	55.5
XII	5.475	6.575	1.1	51.7
XII	6.575	6.625	0.05	55.6
XII	6.625	6.894	0.269	52.3

Additional Table T16. Coordinates, sizes and GC levels of *P. knowlesi* segments.

Chr	Start	End	Size, Mb	GC,%
1	0	0.05	0.05	35.5
1	0.05	0.25	0.2	39.9
1	0.25	0.50	0.25	43.6
1	0.50	0.839	0.339	38.3
2	0	0.1	0.1	37.9
2	0.1	0.525	0.425	43.6
2	0.525	0.675	0.15	40.1
2	0.675	0.725	0.05	33.2
3	0	0.075	0.075	37.6
3	0.075	0.50	0.425	42.8
3	0.50	0.625	0.125	37.4
3	0.625	0.875	0.25	44.7
3	0.875	0.973	0.098	37.9
4	0	0.05	0.05	37.1
4	0.05	0.375	0.325	43.4
4	0.375	0.425	0.05	36.4
4	0.425	0.725	0.3	42.7
4	0.725	0.785	0.06	40.1
5	0	0.1	0.1	34.8
5	0.1	0.15	0.05	39.3
5	0.15	0.55	0.4	42.4
5	0.55	0.600	0.05	40.1
5	0.600	1.225	0.625	42.3
6	0	0.9	0.9	42.7
6	0.9	1.0	0.1	39.8
6	1.0	1.053	0.053	35.1
7	0	0.250	0.25	38.3
7	0.250	1.275	1.025	42.2
7	1.275	1.325	0.05	37.7
7	1.325	1.45	0.125	45.1
7	1.45	1.496	0.046	38.2
8	0	0.075	0.075	34.9
8	0.075	0.7	0.625	39.5
8	0.7	1.7	1	42.7
8	1.7	1.77	0.07	37.2
9	0	0.125	0.125	38.7
9	0.125	0.275	0.15	42.3
9	0.275	0.325	0.05	38.9
9	0.325	0.4	0.075	41.9
9	0.4	0.475	0.075	39.7

9	0.475	0.8	0.325	42.8
9	0.8	0.85	0.05	36.2
9	0.85	1.325	0.475	42.4
9	1.325	1.750	0.425	40.0
9	1.750	1.925	0.175	41.5
9	1.925	2.05	0.125	39.8
9	2.05	2.10	0.05	35.5
9	2.10	2.147	0.047	38.7
10	0	0.1	0.1	35.0
10	0.1	0.575	0.475	39.2
10	0.575	1.0	0.425	43.0
10	1.0	1.05	0.05	37.5
10	1.05	1.375	0.325	43.7
10	1.375	1.425	0.05	37.7
10	1.425	1.485	0.06	35.3
11	0	0.075	0.075	34.8
11	0.075	1.525	1.45	39.9
11	1.525	1.775	0.25	43.0
11	1.775	1.85	0.075	37.7
11	1.85	2.3	0.45	42.7
11	2.3	2.373	0.073	35.2
12	0	0.05	0.05	34.6
12	0.05	0.15	0.1	39.6
12	0.15	0.2	0.05	41.5
12	0.2	0.675	0.475	40.4
12	0.675	0.750	0.075	42.0
12	0.750	0.8	0.05	40.5
12	0.8	1.05	0.25	43.2
12	1.05	1.1	0.05	37.0
12	1.1	1.425	0.325	43.0
12	1.425	2.5	1.075	39.4
12	2.5	2.55	0.05	41.7
12	2.55	3.00	0.45	38.9
12	3.00	3.128	0.128	34.7
13	0	0.05	0.05	33.4
13	0.05	0.1	0.05	38.5
13	0.1	0.25	0.15	41.8
13	0.25	0.375	0.125	39.4
13	0.375	0.675	0.3	41.5
13	0.675	0.750	0.075	40.3
13	0.750	0.825	0.075	43.0
13	0.825	0.9	0.075	35.4
14	0	0.125	0.125	34.7

14	0.125	1.825	1.7	38.6
14	1.825	2.125	0.3	43.1
14	2.125	2	0.05	38.5
14	2	2.35	0.175	43.9
14	2.35	2.425	0.075	39.4
14	2.425	2.575	0.15	42.4
14	2.575	3.159	0.584	37.9

Additional Table T17. Coordinates, sizes and GC levels of *P. berghei* segments.

Chr	Start	End	Size, Mb	GC,%
1	0	0.1	0.1	22.8
1		0.38	0.38	21.4
2	0	0.075	0.075	22.7
2	0.075	0.125	0.05	21
2	0.125	0.2	0.075	22.9
2	0.2	0.5	0.3	21.5
2	0.5	0.55	0.05	25.3
2	0.55	0.64	0.09	21.9
3	0	0.1	0.1	21.1
3	0.1	0.25	0.15	22.8
3	0.25	0.375	0.125	20.9
3	0.375	0.45	0.075	23.3
3	0.45	0.6	0.15	21.4
4	0	0.425	0.425	22.3
4	0.425	0.475	0.05	20.3
4	0.475	0.725	0.25	25.3
5	0	0.1	0.1	22.5
5	0.1	0.3	0.2	21.3
5	0.3	0.35	0.05	23.8
5	0.35	0.625	0.275	21.7
5	0.625	0.725	0.1	24.6
5	0.725	0.92	0.195	21.9
6	0	0.175	0.175	21.2
6	0.175	0.3	0.125	22.7
6	0.3	0.525	0.225	21.5
6	0.525	0.94	0.415	22.4
7	0	0.65	0.65	22
7	0.65	0.82	0.17	20.9
8	0	0.225	0.225	21.7
8	0.225	0.3	0.075	24.3
8	0.875	1.175	0.3	21.8
8	1.175	1.225	0.05	19.9
8	1.225	1.36	0.135	21.4
9	0	0.925	0.925	21.9
9	0.925	0.975	0.05	23.5
9	0.975	1.175	0.2	21.9
9	1.175	1.250	0.075	23.4
9	1.250	1.640	0.39	22
10	0	0.05	0.05	22.4
10	0.05	0.425	0.375	23.9

10	0.425	1.582	1.157	21.8
11	0	0.9	0.9	21.7
11	0.9	0.975	0.075	23.5
11	0.975	1.35	0.375	22.1
11	1.35	1.45	0.1	23.8
11	1.45	2	0.281	21.9
12	0	0.225	0.225	21.9
12	0.225	0.275	0.05	23.4
12	0.275	1.3	1.025	22.1
12	1.3	1.35	0.05	20.4
12	1.35	1.77	0.42	22
13	0	0.6	0.6	22.4
13	0.6	0.650	0.05	23.7
13	0.650	0.7	0.05	22.3
13	0.7	0.8	0.1	23.8
13	0.8	2.504	1.704	22.1
14	0	0.425	0.425	23
14	0.425	0.550	0.125	21.5
14	0.550	2.458	1.908	22.2

Additional Table T18. Coordinates, sizes and GC levels of *P. chabaudi* segments.

Chr	Start	End	Size, Mb	GC,%
1	0	0.175	0.175	23.5
1	0.175	0.3	0.125	24.6
1	0.3	0.582	0.282	23.6
2	0	0.4	0.4	24.2
2	0.4	0.45	0.05	26.2
2	0.45	0.499	0.049	23.1
3	0	0.175	0.175	23.6
3	0.175	0.250	0.075	25.6
3	0.250	0.374	0.124	24.1
4	0	0.1	0.1	23
4	0.1	0.2	0.1	24.3
4	0.2	0.35	0.15	23.2
4	0.35	0.425	0.075	24.7
4	0.425	0.794	0.369	23.8
5	0	0.125	0.125	23.3
5	0.125	0.2	0.075	24.8
5	0.2	0.936	0.736	23.8
6	0	0.075	0.075	24.7
6	0.075	0.8	0.725	23.6
6	0.8	0.917	0.117	24.2
7	0	0.5	0.5	23.7
7	0.5	0.6	0.1	22.8
7	0.6	1.075	0.475	24.2
7	1.075	1.169	0.094	22
8	0	0.25	0.25	22.7
8	0.25	1.36	1.11	23.7
9	0	0.475	0.475	23.8
9	0.475	0.575	0.1	24.8
9	0.575	1.345	0.77	23.4
10	0	0.6	0.6	24.1
10	0.6	0.679	0.079	25.3
10	0.679	1.635	0.956	23.4
11	0	0.25	0.25	23.6
11	0.25	0.325	0.075	25.2
11	0.325	1.721	1.396	23.4
12	0	0.950	0.95	23.3
12	0.950	1.175	0.225	24.2
12	1.175	1.68	0.505	23.2
13	0	2.617	2.617	23.6
14	0	2.515	2.515	23.4

Additional Table T19. Coordinates, sizes and GC levels of *G. theta* segments*.

Chr	Start	End	Size, Mb	GC,%
**1**	0	0.196	0.196	26
**2**	0	0.15	0.15	25.2
**2**	0.15	0.181	0.031	39.6
**3**	0	0.174	0.174	27

*We also analyzed the cryptomonad *Guillardia theta* genome but the three chromosomes' sequences were not complete, being very full of gaps

Additional Table T20. Coordinates, sizes and GC levels of *D. discoideum* segments.

Chr	Start	End	Size, Mb	GC,%
1	0	0.15	0.15	35.3
1	0.15	0.225	0.075	31.6
1	0.225	4.92	4.695	27.7
2	0	8.484	8.484	27.5
3	0	2.575	2.575	28.0
3	2.575	2.625	0.05	26.1
3	2.625	6.357	3.732	27.5
4	0	5.3	5.3	27.6
4	5.3	5.45	0.15	25.3
5	0	4.5	4.5	27.7
5	4.5	4.6	0.1	25.4
5	4.6	5.125	0.525	27.7
6	0	0.325	0.325	28.7
6	0.325	0.375	0.05	25.1
6	0.375	0.85	0.475	27.6
6	0.85	0.9	0.05	32.5
6	0.9	1.95	1.05	27.9
6	1.95	2	0.05	31.3
6	2	3.225	1.225	28.3
6	3.225	3.275	0.05	25.6
6	3.275	3.425	0.15	37.3
6	3.425	3.475	0.05	24.7
6	3.475	3.6	0.125	27.0
